# Delay-Induced Hopf Bifurcation and Entropy-Based Distributional Uncertainty in a Stochastic Time-Delay Pheromone Feedback Model of Ant Foraging Dynamics

**DOI:** 10.3390/e28070751

**Published:** 2026-07-01

**Authors:** Jiaxin Zhu, Luyan Wang, Qiubao Wang

**Affiliations:** Department of Mathematics and Physics, Shijiazhuang Tiedao University, Shijiazhuang 050043, China; 20234632@student.stdu.edu.cn (J.Z.); 1202562006@student.stdu.edu.cn (L.W.)

**Keywords:** stochastic delay system, Hopf bifurcation, time delay, noise, Shannon entropy, Jensen–Shannon divergence, ant foraging system

## Abstract

This study proposes a stochastic time-delay pheromone feedback model to describe ant foraging dynamics, and investigates how response delays and environmental noise jointly induce stochastic oscillations and reorganize the system’s probabilistic structure. By employing near-Hopf center-mode projection and stochastic averaging, we derive the first-order stochastic amplitude equation and analyze the stochastic dynamical properties near the deterministic delay-induced Hopf bifurcation. Subsequently, normalized Shannon entropy and Jensen–Shannon divergence, computed relative to a pre-Hopf stochastic stationary reference distribution, are used to quantify uncertainty expansion and distributional reorganization in the stationary amplitude distribution and reconstructed state-variable distributions. The analytical results are supported by numerical simulations, which indicate that response delay primarily determines the transition from stable foraging to oscillatory behavior, while noise intensity mainly affects the dispersion and uncertainty of the amplitude distribution. Information-theoretic metrics further reveal noise-induced uncertainty growth and delay-induced probabilistic restructuring. This study elucidates the stability regulation mechanisms of ant foraging systems under stochastic conditions from a combined dynamical and information-theoretic perspective, and provides a theoretical reference for the design of delayed feedback in swarm intelligence systems.

## 1. Introduction

Ant colonies exhibit remarkable collective foraging capabilities through decentralized interactions among individuals. Without centralized control, ants rely on local communication and environmental feedback to accomplish tasks such as food searching, forager allocation, traffic flow regulation, and path network maintenance [1,2,3,4]. Among these mechanisms, pheromones play a key role in linking individual behavior to group-level organization. Furthermore, there is a mixed feedback loop between ant movement across different behavioral compartments and pheromone concentrations along their paths [5,6]. Pheromone deposition serves as a source of positive feedback, reinforcing successful foraging paths, whereas pheromone evaporation and limited individual responsiveness provide negative feedback that enables adaptive regulation [7,8,9]. Consequently, from the perspective of complex systems, ant foraging represents a typical example of self-organization, collective decision-making, and swarm intelligence [10,11,12,13].

In 2015, Udiani et al. developed a three-compartment model of collective foraging in harvester ants, which revealed how local individual interactions induce the formation of a robust collective foraging regulatory system through feedback mechanisms [14]. In 2016, Ryan constructed a PDE-ODE coupled model describing collective foraging behavior in ants, integrating foraging ants, returning ants, and pheromone concentration into a unified framework. This model revealed the dynamical mechanisms by which ant colonies transition from disordered individual motion to ordered collective motion under pheromone regulation [15]. In 2018, Pagliara et al. described the foraging regulation of harvester ants as a closed-loop excitable system. By establishing a low-dimensional dynamical model, they revealed the principle by which returning foragers and pre-emergent ants regulate foraging activity through feedback mechanisms [16]. Since 2021, Feng et al. have made a series of contributions to the study of collective foraging dynamics in social insects under stochastic and dynamic conditions. Their research has progressively incorporated resource constraints, environmental fluctuations, and nonlinear recruitment mechanisms into deterministic and stochastic modeling frameworks, revealing the effects of noise intensity, task demands, and environmental variability on group sustainability as well as dynamic behaviors such as bifurcation and stability transitions [17,18,19]. However, these studies have mainly focused on dynamical stability, bifurcation mechanisms, or stochastic persistence, while the information-theoretic characterization of uncertainty and probabilistic structural reorganization in delayed stochastic ant foraging systems remains insufficiently explored.

Time delays are important factors influencing the dynamic behavior of biological and ecological systems [20]. Time delays can alter the stability of equilibrium points and induce oscillations in nonlinear systems via Hopf bifurcations [21]. Zhang, Liu, and Wei established a time-delay mutualistic system with a phase structure based on the mutualistic relationship between leaf-cutting ants and fungal gardens and investigated its stability and Hopf bifurcations [22]. During ant foraging, the response of ants to changes in pheromone concentration is not instantaneous but involves a certain delay. Therefore, incorporating time delays into ant foraging models not only improves the description of feedback regulation but also provides a mechanism through which collective dynamics and probabilistic organization may undergo qualitative transitions.

Stochastic influences are also inevitable in natural foraging environments. Ant foraging behavior is influenced by factors such as individual differences, environmental fluctuations, uncertainties in pheromone perception, and path disruptions [23,24]. Deterministic models may fail to capture the uncertainty and variability observed in real collective foraging environments. Compared to deterministic equations, stochastic equations can more accurately reflect real-world scenarios [25,26]. Dodoková et al. developed a stochastic model of ant trail formation and maintenance, revealing the role of random motion and pheromone interactions in trail exploration and maintenance [27]. These random factors can be approximated by Gaussian white noise and subsequently incorporated into a deterministic ant foraging model to investigate the effects of noise on the system’s dynamic behavior, as well as the propagation of uncertainty and stochastic transitions [28].

In stochastic dynamical systems, the transition from small fluctuations around an equilibrium to distributed oscillatory states is not fully characterized by the most probable amplitude alone because the mode only describes the peak location of the stationary density. Variance and kurtosis provide useful moment-based information about dispersion and tail behavior, but they do not directly characterize the global redistribution of probability mass. Since the present study focuses on uncertainty expansion and probabilistic structural reorganization of the stationary amplitude distribution, information-theoretic measures are more appropriate for this purpose. In particular, Shannon entropy provides a natural framework for characterizing uncertainty and probabilistic organization in stochastic dynamical systems [29]. In addition, the Jensen–Shannon divergence provides a symmetric measure for comparing probability distributions [30]. These measures are particularly important for delayed stochastic foraging systems, in which response delays and environmental noise can not only alter system stability and oscillation amplitude but also reshape their underlying probabilistic structure. In this study, Hopf bifurcation refers to the delay-induced transition of the deterministic noise-free subsystem, whereas the stochastic bifurcation analyzed in the reduced amplitude equation is a P-bifurcation of the stationary probability density rather than a D-bifurcation.

Based on the model proposed in [31], this paper investigates the effects of response delay and Gaussian white noise on ant foraging systems. The main contributions of this paper are summarized as follows:

(i) A delayed pheromone feedback ant-foraging model with additive Gaussian perturbation is formulated.
(ii) The delay-induced Hopf threshold is derived from the characteristic equation and verified numerically.
(iii) A near-Hopf stochastic amplitude equation is obtained through center-mode projection and stochastic averaging [32,33].
(iv) An entropy-based characterization framework is incorporated into a stochastic P-bifurcation analysis, in which normalized Shannon entropy and Jensen–Shannon divergence, evaluated relative to a pre-Hopf stochastic stationary reference distribution, are used to quantify uncertainty expansion and probabilistic structural reorganization induced by response delay and noise.

This study provides an information-theoretic perspective on stochastic ant foraging dynamics and delayed feedback in swarm intelligence systems.

The structure of this paper is organized as follows: Section 2 establishes a stochastic delayed ant foraging model, determines the coexistence equilibrium, and analyzes its local stability and delay-induced Hopf bifurcation. Subsequently, the existence conditions for Hopf bifurcation are derived, and a stability analysis is performed. Section 3 derives a leading-order stochastic amplitude equation through near-Hopf center-mode projection and stochastic averaging and further analyzes stochastic P-bifurcation together with entropy-based uncertainty characterization of the stationary amplitude distribution. Section 4 verifies the theoretical analysis through numerical simulations and investigates how response delay and noise intensity influence stochastic oscillations, uncertainty expansion, and probabilistic structural reorganization in the ant foraging system. Finally, Section 5 summarizes the main conclusions of this study and discusses potential future research directions.

## 2. Equilibrium, Stability and Delay-Induced Hopf Bifurcation

Building on [31], we incorporate response delay and Gaussian white noise into the pheromone-feedback foraging model, resulting in the following dynamical system:

(1) $$\begin{array}{cc} \dot{R} & =\alpha \left(1-R\right)-\beta \frac{M}{N}RS;\\ \dot{S} & =\gamma \left(1-S\right)-\beta \frac{N}{M}RS;\\ \dot{p} & =-\mu p+\nu \left[\gamma M(1-S)+\beta NRS\right];\\ d\gamma (t) & =\frac{-\gamma \left(t\right)+f\left(p(t-\tau )\right)+\gamma_{0}}{\tau_{\gamma }}dt+\frac{\sigma }{\tau_{\gamma }}dW_{t};\\ \; & R+I=1,\;S+F=1. \end{array}$$

In this context, *R* denotes the proportion of receiver ants in the nest population; *I* denotes the proportion of internal ants in the nest population; *S* denotes the proportion of supplier ants within the trail population; *F* denotes the proportion of foraging ants actively searching for food on the trail within the trail population. *N* denotes the number of nest ants, *M* denotes the number of trail ants, and α denotes the rate at which internal ants convert to receiver ants at the nest entrance. β denotes the supplier-receiver interaction rate coefficient. μ denotes the pheromone evaporation rate. ν denotes the pheromone deposition rate. *p* denotes the pheromone concentration on the trail near the nest entrance, and γ denotes the return-to-nest rate. $\gamma_{0}$ denotes the baseline rate of γ, representing the baseline return-to-nest rate. $\tau_{\gamma }$ denotes the characteristic timescale for the return rate γ to respond to changes in pheromone concentration.

The delay parameter τ is interpreted as an effective dimensionless response delay associated with pheromone-mediated feedback. Let $t_{\mathrm{phys}}$ denote the dimensional physical time and let $T_{\gamma }$ be the physical counterpart of $\tau_{\gamma }$, representing the characteristic time over which the return-to-nest rate γ responds to pheromone concentration changes. By introducing the dimensionless time $t=t_{\mathrm{phys}}/T_{\gamma }$, the dimensional delay $T_{\mathrm{delay}}$ and the dimensionless delay τ satisfy

$$\tau =\frac{T_{\mathrm{delay}}}{T_{\gamma }},\;T_{\mathrm{delay}}=\tau T_{\gamma }.$$

Therefore, the dimensionless delay used in the model can be converted into physical time once the characteristic response time $T_{\gamma }$ is specified.

**Figure 1:**
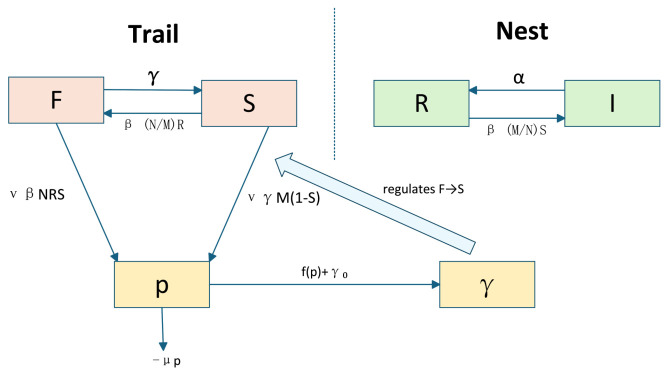
Schematic diagram of the model.

$W_{t}$ denotes a standard one-dimensional Brownian motion. Equivalently, the formal white noise $\xi \left(t\right)=dW_{t}/dt$ satisfies $E\left[\xi \left(t\right)\right]=0$ and $E\left[\xi \left(t\right)\xi \left(s\right)\right]=\delta \left(t-s\right)$. σ is a sufficiently small noise-intensity parameter. Here, the additive perturbation in the equation governing γ(t) represents unresolved fluctuations in the return-to-nest response, arising from individual variability, local environmental disturbances, and uncertainty in pheromone perception. Since the analysis is local near the coexistence equilibrium, these fluctuations are assumed to have an intensity that is approximately independent of the current state variables; in particular, the noise intensity is not taken to scale with the pheromone concentration or the return rate itself. Thus, additive Gaussian white noise provides a first-order local approximation, whereas $f\left(p(t-\tau )\right)+\gamma_{0}$ describes the deterministic mean response to delayed pheromone feedback. The function *f* describes the dependence of the return rate on pheromone concentration. Since *f* must satisfy the biological requirement that an increase in pheromone concentration raises the return rate and eventually reaches saturation, we choose *f* to be the logistic function $f\left(p\right)=\frac{\kappa }{1+\exp\left(-\eta \left(p-p_{0}\right)\right)}$, where κ, $p_{0}$, and η are positive parameters. A schematic diagram of this model is shown in Figure 1.

It is worth noting that the modeling approach adopted in this study is not limited to ant foraging dynamics. Similar nonlinear feedback structures with time delays have also been widely applied in models of real-world ecological interactions, such as predator–prey systems involving fishing, fear effects, indirect predation, and delayed responses [34,35]. These studies indicate that delayed nonlinear interactions can trigger stability transitions, periodic oscillations, and complex bifurcation phenomena. Unlike predator–prey models, the variables in this system describe behavioral compartments and pheromone-mediated feedback, rather than population densities between species. Nevertheless, their shared mathematical characteristics—including nonlinear feedback, response delays, random perturbations, and bifurcation-induced changes—suggest that the entropy-based framework developed in this study can provide a useful reference for related delayed stochastic biological models.

To characterize the fundamental dynamical behavior of this system under long-term evolution, we first examine the existence of its equilibrium states and their local stability. At an equilibrium $E^{*}=\left(R^{*},\;S^{*},\;p^{*},\;\gamma^{*}\right)$, the delay term satisfies $p\left(t-\tau \right)=p^{*}$, and the stochastic perturbation has zero mean. Therefore, the deterministic equilibrium is determined by

(2) $$\begin{array}{cc} \; & \alpha \left(1-R^{*}\right)-\beta \frac{M}{N}R^{*}S^{*}=0;\\ \; & \gamma^{*}\left(1-S^{*}\right)-\beta \frac{N}{M}R^{*}S^{*}=0;\\ \; & -\mu p^{*}+\nu \left[\gamma^{*}M\left(1-S^{*}\right)+\beta NR^{*}S^{*}\right]=0;\\ \; & -\gamma^{*}+f\left(p^{*}\right)+\gamma_{0}=0. \end{array}$$

In the following analysis, we assume that the system admits a positive coexistence equilibrium $E^{*}$ satisfying the above equations. Let $r\left(t\right)=R\left(t\right)-R^{*}$; $s\left(t\right)=S\left(t\right)-S^{*}$; q$\left(t\right)=p\left(t\right)-p^{*}$; $g\left(t\right)=\gamma \left(t\right)-\gamma^{*},$ where *r*, *s*, *q*, *g* denote the small deviations of the system states relative to the equilibrium point. Substituting the above transformations into the original system and performing a Taylor expansion, followed by linearization in the neighborhood of $E^{*}$, yields the following vector form:

Linearized system:
  (3) $$\frac{dX\left(t\right)}{dt}=AX\left(t\right)+BX\left(t-\tau \right).$$
Local system with nonlinear remainder:
  (4) $$\frac{dX\left(t\right)}{dt}=AX\left(t\right)+BX\left(t-\tau \right)+F\left(X_{t}\right).$$

$$A=\left(\begin{array}{cccc} a_{11} & a_{12} & 0 & 0\\ a_{21} & a_{22} & 0 & a_{24}\\ a_{31} & a_{32} & a_{33} & a_{34}\\ 0 & 0 & 0 & a_{44} \end{array}\right),$$

$$B=\left(\begin{array}{cccc} 0 & 0 & 0 & 0\\ 0 & 0 & 0 & 0\\ 0 & 0 & 0 & 0\\ 0 & 0 & b_{43} & 0 \end{array}\right),$$

where

$$\begin{array}{cc} a_{11} & =-\alpha -\beta \frac{M}{N}S^{*};a_{12}=-\beta \frac{M}{N}R^{*};a_{21}=-\beta \frac{N}{M}S^{*};\\ a_{22} & =-\gamma^{*}-\beta \frac{N}{M}R^{*};a_{24}=1-S^{*};a_{31}=\nu \beta NS^{*};\\ a_{32} & =\nu \left(-\gamma^{*}M+\beta NR^{*}\right);a_{33}=-\mu ;a_{34}=\nu M\left(1-S^{*}\right);\\ \; & \;a_{44}=-\frac{1}{\tau_{\gamma }};\;b_{43}=\frac{f^{\prime }\left(p^{*}\right)}{\tau_{\gamma }}. \end{array}$$

For the deterministic noise-free subsystem, the characteristic equation can be expressed in the following determinant form: $\left|\lambda I-A-Be^{-\lambda \tau }\right|=0$.

Here, we denote the determinant of the above characteristic matrix as $\Delta \left(\lambda ,\tau \right)$:

(5) $$\begin{array}{cc} \Delta (\lambda ,\tau )= & \left(\lambda +\frac{1}{\tau_{\gamma }}\right)\left(\lambda +\mu \right)\left[\left(\lambda +\alpha +\beta \frac{M}{N}S^{*}\right)\left(\lambda +\gamma^{*}+\beta \frac{N}{M}R^{*}\right)-\beta^{2}R^{*}S^{*}\right]\\ \; & +e^{-\lambda \tau }\frac{f^{\prime }\left(p^{*}\right)}{\tau_{\gamma }}\{\left(\lambda +\alpha +\beta \frac{M}{N}S^{*}\right)[-\nu M\left(1-S^{*}\right)\lambda \\ \; & \;+\left(-\gamma^{*}-\beta \frac{N}{M}R^{*}\right)\nu M\left(1-S^{*}\right)-\nu \left(-\gamma^{*}M+\beta NR^{*}\right)\left(1-S^{*}\right)]\\ \; & \;-\beta \frac{M}{N}R^{*}\left[-\beta \frac{N}{M}S^{*}\nu M\left(1-S^{*}\right)-\nu \beta NS^{*}\left(1-S^{*}\right)\right]\}. \end{array}$$

For convenience, the characteristic equation can be rewritten as

(6) $$\Delta \left(\lambda ,\tau \right)=P\left(\lambda \right)+b_{43}Q\left(\lambda \right)e^{-\lambda \tau }=0.$$

where

$$\begin{array}{cc} \; & P\left(\lambda \right)=\lambda^{4}-m_{1}\lambda^{3}+m_{2}\lambda^{2}+m_{3}\lambda +m_{4};\\ \; & Q\left(\lambda \right)=-n_{1}\lambda^{2}+n_{2}\lambda +n_{3}. \end{array}$$

Substituting λ=iω and writing $P\left(i\omega \right)=P_{R}+iP_{I}$ and $Q\left(i\omega \right)=Q_{R}+iQ_{I}$, we obtain

$$\begin{array}{cc} \; & P_{R}=\omega^{4}-m_{2}\omega^{2}+m_{4};\;P_{I}=m_{1}\omega^{3}+m_{3}\omega ;\\ \; & Q_{R}=n_{1}\omega^{2}+n_{3};\;\;\;\;Q_{I}=n_{2}\omega . \end{array}$$

Since $e^{-i\omega \tau }=\cos\omega \tau -i\sin\omega \tau$, separating the real and imaginary parts gives:

(7) $$\begin{array}{cc} \; & P_{R}+b_{43}\left(Q_{R}\cos\omega \tau +Q_{I}\sin\omega \tau \right)=0;\\ \; & P_{I}+b_{43}\left(Q_{I}\cos\omega \tau -Q_{R}\sin\omega \tau \right)=0. \end{array}$$

Solving the linear system (7), we obtain the following result:

(8) $$\sin\omega \tau =\frac{\left(m_{1}\omega^{3}+m_{3}\omega \right)\left(n_{1}\omega^{2}+n_{3}\right)-\left(\omega^{4}-m_{2}\omega^{2}+m_{4}\right)n_{2}\omega }{b_{43}\left({\left(n_{1}\omega^{2}+n_{3}\right)}^{2}+n_{2}^{2}\omega^{2}\right)};$$

(9) $$\;\cos\omega \tau =\frac{-\left(\omega^{4}-m_{2}\omega^{2}+m_{4}\right)\left(n_{1}\omega^{2}+n_{3}\right)-\left(m_{1}\omega^{3}+m_{3}\omega \right)n_{2}\omega }{b_{43}\left({\left(n_{1}\omega^{2}+n_{3}\right)}^{2}+n_{2}^{2}\omega^{2}\right)}.$$

where

$$\begin{array}{cc} \; & m_{1}=-\left(\alpha +\gamma^{*}+\mu +\frac{1}{\tau_{\gamma }}+\beta \frac{M}{N}S^{*}+\beta \frac{N}{M}R^{*}\right);\\ \; & m_{2}=\alpha \gamma^{*}+\alpha \beta \frac{N}{M}R^{*}+\beta \frac{M}{N}S^{*}\gamma^{*}+\mu \left(\alpha +\gamma^{*}+\beta \frac{M}{N}S^{*}+\beta \frac{N}{M}R^{*}\right)\\ \; & +\frac{\alpha +\gamma^{*}+\beta \frac{M}{N}S^{*}+\beta \frac{N}{M}R^{*}+\mu }{\tau_{\gamma }};\\ \; & m_{3}=\left(\mu +\frac{1}{\tau_{\gamma }}\right)\left(\alpha \gamma^{*}+\alpha \beta \frac{N}{M}R^{*}+\beta \frac{M}{N}S^{*}\gamma^{*}\right)\\ \; & +\frac{\mu }{\tau_{\gamma }}\left(\alpha +\gamma^{*}+\beta \frac{M}{N}S^{*}+\beta \frac{N}{M}R^{*}\right);\\ \; & m_{4}=\frac{\mu }{\tau_{\gamma }}\left(\alpha \gamma^{*}+\alpha \beta \frac{N}{M}R^{*}+\beta \frac{M}{N}S^{*}\gamma^{*}\right);\\ \; & n_{1}=\nu M\left(1-S^{*}\right);\\ \; & n_{2}=-\nu \left(1-S^{*}\right)\left(M\alpha +\beta \frac{M^{2}}{N}S^{*}+2\beta NR^{*}\right);\\ \; & n_{3}=-2\nu \alpha \beta NR^{*}\left(1-S^{*}\right). \end{array}$$

Squaring both equations and adding them together, we get:

(10) $$\omega^{8}+d_{1}\omega^{6}+d_{2}\omega^{4}+d_{3}\omega^{2}+d_{4}=0.$$

Let $x=\omega^{2}$; then the above equation is equivalent to

(11) $$L\left(x\right)=x^{4}+d_{1}x^{3}+d_{2}x^{2}+d_{3}x+d_{4}=0.$$

where

$$\begin{array}{cc} \; & d_{1}=m_{1}^{2}-2m_{2};\\ \; & d_{2}=m_{2}^{2}+2m_{1}m_{3}+2m_{4}-b_{43}^{2}n_{1}^{2};\\ \; & d_{3}=m_{3}^{2}-2m_{2}m_{4}-2b_{43}^{2}n_{1}n_{3}-b_{43}^{2}n_{2}^{2};\\ \; & d_{4}=m_{4}^{2}-b_{43}^{2}n_{3}^{2}. \end{array}$$

Since $\lim_{x\to \infty }L\left(x\right)=\infty$, if condition H(1): $\exists x^{*}>0,\;L\left(x^{*}\right)<0$ holds, then Equation (11) has at least one positive real root $x_{i}\left(1\leq i\leq 4\right)$; therefore, Equation (10) has at least one positive real root $\omega_{i}=\sqrt{x_{i}}$. Let us denote the right-hand side of the expression $\sin\omega_{i}\tau$ by $S_{i}$ and the right-hand side of the expression $\cos\omega_{i}\tau$ by $C_{i}$. Then Equations (8) and (9) can be transformed as follows:

$$\theta_{i}=\mathrm{atan}2\left(S_{i},C_{i}\right).$$

Taking $\theta_{i}\in \left[0,\;2\pi \right)$, the corresponding critical delays are given by

$$\tau_{i}^{\left(j\right)}=\frac{\theta_{i}+2j\pi }{\omega_{i}},\;j=0,1,2,\ldots .$$

Therefore, the first positive critical delay is defined as

$$\tau_{0}=\min_{\begin{array}{c} i=1,\ldots ,4\\ j\geq 0,\;\tau_{i}^{\left(j\right)}>0 \end{array}}\tau_{i}^{\left(j\right)}=\min_{\begin{array}{c} i=1,\ldots ,4\\ j\geq 0,\;\tau_{i}^{\left(j\right)}>0 \end{array}}\frac{\theta_{i}+2j\pi }{\omega_{i}}.$$

Let $\left(i_{0},\;j_{0}\right)$ be the index pair attaining this minimum. Then

$$\tau_{0}=\tau_{i_{0}}^{\left(j_{0}\right)},\;\omega_{0}=\omega_{i_{0}}.$$

When $\tau =\tau_{0}$, the characteristic Equation (5) has a pair of purely imaginary roots $\lambda =\pm i\omega_{0}\left(\omega_{0}>0\right)$.

Here, we make the following assumption:

$$\begin{array}{cc} \; & H\left(2\right):I=\left(-4n_{1}\omega^{6}-3m_{1}n_{2}\omega^{4}-4n_{3}\omega^{4}+2m_{2}n_{1}\omega^{4}-m_{3}n_{2}\omega^{2}+2m_{2}n_{3}\omega^{2}\right)\\ \; & \left(-n_{1}\omega^{6}-n_{3}\omega^{4}+n_{1}m_{2}\omega^{4}-m_{1}n_{2}\omega^{4}-m_{3}n_{2}\omega^{2}+m_{2}n_{3}\omega^{2}-m_{4}n_{1}\omega^{2}-m_{4}n_{3}\right)\\ \; & -\left(-2m_{2}n_{2}\omega^{3}+4n_{2}\omega^{5}-3m_{1}n_{1}\omega^{5}-3m_{1}n_{3}\omega^{3}-m_{3}n_{1}\omega^{3}-m_{3}n_{3}\omega \right)\\ \; & \left(m_{1}n_{1}\omega^{5}+m_{1}n_{3}\omega^{3}+m_{3}n_{1}\omega^{3}+m_{3}n_{3}\omega -n_{2}\omega^{5}+m_{2}n_{2}\omega^{3}-m_{4}n_{2}\omega \right)\\ \; & -b_{43}^{2}\left(n_{2}^{2}\omega^{2}+2n_{1}^{2}\omega^{4}+2n_{1}n_{3}\omega^{2}\right)\left[n_{2}^{2}\omega^{4}+{\left(n_{1}\omega^{2}+n_{3}\right)}^{2}\right]\neq 0. \end{array}$$

**Theorem** **1.** *If H(1) yields a simple pair of purely imaginary roots $\pm i\omega_{0}$ at $\tau =\tau_{0}$, all other characteristic roots have nonzero real parts, and H(2) gives the transversality condition, then the coexistence equilibrium undergoes a Hopf bifurcation at $\tau =\tau_{0}$.*

**Proof.**  Taking the derivative of both sides of Equation (5) with respect to τ, the Hopf transversality condition is as follows:

(12) $${\left(\frac{d\lambda }{d\tau }\right)}^{-1}=\frac{4\lambda^{3}-3m_{1}\lambda^{2}+2m_{2}\lambda +m_{3}}{b_{43}e^{-\lambda \tau }\left(-n_{1}\lambda^{3}+n_{2}\lambda^{2}+n_{3}\lambda \right)}-\frac{2n_{1}\lambda -n_{2}}{-n_{1}\lambda^{3}+n_{2}\lambda^{2}+n_{3}\lambda }-\frac{\tau }{\lambda }.$$

Redλdτ−1τ=τ0=[−4n1ω6−3m1n2ω4−4n3ω4+2m2n1ω4−m3n2ω2+2m2n3ω2−n1ω6−n3ω4+n1m2ω4−m1n2ω4−m3n2ω2+m2n3ω2−m4n1ω2−m4n3−4n2ω5−3m1n1ω5−2m2n2ω3−3m1n3ω3−m3n1ω3−m3n3ωm1n1ω5−n2ω5+m1n3ω3+m2n2ω3+m3n1ω3+m3n3ω−m4n2ω−b4322n12ω4+n22ω2+2n1n3ω2n22ω4+n1ω2+n32]/b432n22ω4+n1ω3+n3ω2n22ω4+n1ω2+n32.

Since Re$\left(\frac{d\lambda }{d\tau }\right)$ and Re${\left(\frac{d\lambda }{d\tau }\right)}^{-1}$ have the same sign, if condition H(2) holds, then by Hopf bifurcation theory, a Hopf bifurcation occurs at $\tau =\tau_{0}$, and the theorem is proven. □

The mathematical roles of H(1) and H(2) are to ensure the existence of a positive critical frequency and the transversality of the critical characteristic roots, respectively. For the parameter set used in the numerical simulations, the numerical verification of H(1) and H(2) is provided in Section 4.

## 3. Stochastic Center-Mode Reduction and Entropy-Based P-Bifurcation Analysis

### 3.1. Center-Mode Projection near the Hopf Critical Point

In this section, we derive a near-Hopf stochastic amplitude equation by projecting the stochastic delayed system onto the critical center modes and applying stochastic averaging. Since a fully rigorous stochastic center-manifold construction for delay equations with additive white noise requires additional technical assumptions, the reduction below is used as a leading-order center-mode approximation near the Hopf critical point.

The first step is to perform a center manifold reduction, projecting the system from the infinite-dimensional state space of the delay differential equation (DDE) onto a two-dimensional invariant subspace tangent to the plane spanned by the critical eigenvectors. To study the local dynamics of the system near the Hopf critical point, let $\tau =\tau_{0}+\epsilon \tilde{\tau }$. Then, the system can be represented in $C=C\left(\left[-\tau ,0\right],R^{4}\right)$ as:

(13) $$dx\left(t\right)=\left(L_{\tilde{\tau }}\left(x_{t}\right)+F\left(\tilde{\tau },x_{t}\right)\right)dt+GdW_{t}.$$

where $x_{t}\left(\theta \right)=x\left(t+\theta \right)\in C$, and $L_{\tilde{\tau }}:C\to R^{4},\;F:R\times C\to R^{4}$, and $G\in R^{4}$ are defined as follows:

$$L_{\tilde{\tau }}\left(\rho \right)=A\left(\begin{array}{c} \rho_{1}\left(0\right)\\ \rho_{2}\left(0\right)\\ \rho_{3}\left(0\right)\\ \rho_{4}\left(0\right) \end{array}\right)+B\left(\begin{array}{c} \rho_{1}\left(-\tau \right)\\ \rho_{2}\left(-\tau \right)\\ \rho_{3}\left(-\tau \right)\\ \rho_{4}\left(-\tau \right) \end{array}\right),$$

$$F\left(\tilde{\tau },\rho \right)=\left(\begin{array}{c} -\beta \frac{M}{N}\rho_{1}\left(0\right)\rho_{2}\left(0\right)\\ -\rho_{4}\left(0\right)\rho_{2}\left(0\right)-\beta \frac{N}{M}\rho_{1}\left(0\right)\rho_{2}\left(0\right)\\ -\nu M\rho_{4}\left(0\right)\rho_{2}\left(0\right)+\nu \beta N\rho_{1}\left(0\right)\rho_{2}\left(0\right)\\ \frac{1}{\tau_{\gamma }}\left[f\left(p^{*}+\rho_{3}\left(-\tau \right)\right)-f\left(p^{*}\right)-f^{\prime }\left(p^{*}\right)\rho_{3}\left(-\tau \right)\right] \end{array}\right),$$

$$G=\left(\begin{array}{c} 0\\ 0\\ 0\\ \frac{\sigma }{\tau_{\gamma }} \end{array}\right).$$

By the Riesz representation theorem, there exists a function of bounded variation $\eta \left(\theta ,\tilde{\tau }\right)$ such that $L_{\tilde{\tau }}\phi =\int_{-\tau }^{0}d\eta \left(\theta ,\tilde{\tau }\right)\phi \left(\theta \right)$, where $\eta \left(\theta ,\tilde{\tau }\right)=A\delta \left(\theta \right)+B\delta \left(\theta +\tau \right)$, $\delta \left(\theta \right)=\left\{\begin{array}{c} 0,\;\theta \neq 0\\ 1,\;\theta =0 \end{array}\right..$ For $\phi \in C\left(\left[-\tau ,0\right],R^{4}\right)$, we introduce the following operators:

$$M\left(\tilde{\tau }\right)\phi =\left\{\begin{array}{c} \frac{d\phi \left(\theta \right)}{d\theta },\;\theta \in \left[-\tau ,\;0\right)\\ \int_{-\tau }^{0}d\eta \left(\tilde{\tau },s\right)\phi \left(s\right),\;\theta =0 \end{array}\right.\;,$$

$$N\left(\tilde{\tau }\right)\phi =\left\{\begin{array}{c} 0,\;\theta \in \left[-\tau ,\;0\right)\\ F\left(\tilde{\tau },\phi \right),\;\theta =0 \end{array}\right.\;,$$

$$G\left(\theta \right)=\left\{\begin{array}{c} 0,\;\theta \in \left[-\tau ,\;0\right)\\ G,\;\theta =0 \end{array}\right..$$

Thus, Equation (13) can be equivalently written as

(14) $$dx_{t}=\left[M\left(\tilde{\tau }\right)x_{t}+N\left(\tilde{\tau }\right)x_{t}\right]dt+GdW_{t}.$$

For $\psi \in \hat{C}\left(\left[0,\tau \right],{\left(R^{4}\right)}^{*}\right)$, we define

(15) $$M^{*}\psi =\left\{\begin{array}{c} -\frac{d\psi \left(t\right)}{dt},\;t\in \left(0,\tau \right]\\ \int_{-\tau }^{0}\psi \left(-s\right)d\eta \left(s,0\right),\;t=0 \end{array}\right..$$

where the operator $M^{*}$ is the adjoint of $M\left(0\right)$. For ϕ and ψ, we can define a bilinear pairing

(16) $$\langle \psi \left(t\right),\phi \left(\theta \right)\rangle =\psi \left(0\right)\phi \left(0\right)-\int_{-\tau }^{0}\int_{\xi =0}^{\theta }\psi \left(\xi -\theta \right)d\eta \left(\theta \right)\phi \left(\xi \right)d\xi .$$

where $\eta \left(\theta \right)=\eta \left(\theta ,0\right)$.

Assume $\left(i\omega -A-Be^{-i\omega \tau_{0}}\right)q\left(0\right)=0$, where $q\left(0\right)$ is an eigenvector. Let $q\left(\theta \right)=q\left(0\right)e^{i\omega \theta }$. Combining this with Euler’s formula, we obtain $\Phi \left(\theta \right)=\left(\phi_{1}\left(\theta \right),\phi_{2}\left(\theta \right)\right)$, where $\phi_{1}\left(\theta \right)=Req\left(\theta \right)$, $\phi_{2}\left(\theta \right)=Imq\left(\theta \right)$. We then obtain

$$\Phi \left(\theta \right)=\left(\begin{array}{cc} \phi_{11}\left(\theta \right) & \phi_{12}\left(\theta \right)\\ \phi_{21}\left(\theta \right) & \phi_{22}\left(\theta \right)\\ \phi_{31}\left(\theta \right) & \phi_{32}\left(\theta \right)\\ \phi_{41}\left(\theta \right) & \phi_{42}\left(\theta \right) \end{array}\right),\;-\tau \leq \theta \leq 0$$

$$\begin{array}{cc} \; & \phi_{11}\left(\theta \right)=\cos\omega_{0}\theta ,\\ \; & \phi_{12}\left(\theta \right)=\sin\omega_{0}\theta ,\\ \; & \phi_{21}\left(\theta \right)=\frac{\left(\alpha +\beta \frac{M}{N}S^{*}\right)\cos\omega_{0}\theta -\omega_{0}\sin\omega_{0}\theta }{-\beta \frac{M}{N}R^{*}},\\ \; & \phi_{22}\left(\theta \right)=\frac{\left(\alpha +\beta \frac{M}{N}S^{*}\right)\sin\omega_{0}\theta +\omega_{0}\cos\omega_{0}\theta }{-\beta \frac{M}{N}R^{*}},\\ \; & \phi_{31}\left(\theta \right)=\frac{-E_{R}\cos\omega_{0}\left(\tau +\theta \right)+E_{I}\sin\omega_{0}\left(\tau +\theta \right)}{-\beta \frac{M}{N}R^{*}\left(1-S^{*}\right)\frac{f^{\prime }\left(p^{*}\right)}{\tau_{\gamma }}},\\ \; & \phi_{32}\left(\theta \right)=\frac{-E_{R}\sin\omega_{0}\left(\tau +\theta \right)-E_{I}\cos\omega_{0}\left(\tau +\theta \right)}{-\beta \frac{M}{N}R^{*}\left(1-S^{*}\right)\frac{f^{\prime }\left(p^{*}\right)}{\tau_{\gamma }}},\\ \; & \phi_{41}\left(\theta \right)=\frac{D_{R}\cos\omega_{0}\theta -D_{I}\sin\omega_{0}\theta }{-\beta \frac{M}{N}R^{*}\left(1-S^{*}\right)},\\ \; & \phi_{42}\left(\theta \right)=\frac{D_{R}\sin\omega_{0}\theta +D_{I}\cos\omega_{0}\theta }{-\beta \frac{M}{N}R^{*}\left(1-S^{*}\right)}. \end{array}$$

where

$$\begin{array}{cc} E_{R}= & -\frac{1}{\tau_{\gamma }}\left[\left(-\alpha -\beta \frac{M}{N}S^{*}\right)\left(-\gamma^{*}-\beta \frac{N}{M}R^{*}\right)-\beta^{2}R^{*}S^{*}-\omega_{0}^{2}\right]\\ \; & -\omega_{0}^{2}\left(-\alpha -\beta \frac{M}{N}S^{*}-\gamma^{*}-\beta \frac{N}{M}R^{*}\right),\\ E_{I}= & \frac{\omega_{0}}{\tau_{\gamma }}\left(-\alpha -\beta \frac{M}{N}S^{*}-\gamma^{*}-\beta \frac{N}{M}R^{*}\right)\\ \; & -\omega_{0}\left[\left(-\alpha -\beta \frac{M}{N}S^{*}\right)\left(-\gamma^{*}-\beta \frac{N}{M}R^{*}\right)-\beta^{2}R^{*}S^{*}-\omega_{0}^{2}\right],\\ D_{R}= & \left(-\alpha -\beta \frac{M}{N}S^{*}\right)\left(-\gamma^{*}-\beta \frac{N}{M}R^{*}\right)-\beta^{2}R^{*}S^{*}-\omega_{0}^{2},\\ D_{I}= & -\omega_{0}\left(-\alpha -\beta \frac{M}{N}S^{*}-\gamma^{*}-\beta \frac{N}{M}R^{*}\right). \end{array}$$

Similarly, based on the adjoint relationship between $\Phi \left(\theta \right)$ and $\Psi \left(t\right)$, we obtain $\Psi \left(t\right)=\left(\begin{array}{c} \psi_{1}\left(t\right)\\ \psi_{2}\left(t\right) \end{array}\right)$, where $\psi_{1}\left(t\right)=Req^{*}\left(t\right),\;\psi_{2}\left(t\right)=Imq^{*}\left(t\right)$. We then obtain

$$\Psi \left(t\right)=\left(\begin{array}{cccc} \psi_{11}\left(t\right) & \psi_{12}\left(t\right) & \psi_{13}\left(t\right) & \psi_{14}\left(t\right)\\ \psi_{21}\left(t\right) & \psi_{22}\left(t\right) & \psi_{23}\left(t\right) & \psi_{24}\left(t\right) \end{array}\right),\;0\leq t\leq \tau$$

$$\begin{array}{cc} \; & \psi_{11}\left(t\right)=\alpha_{1}\cos\omega_{0}t-\beta_{1}\sin\omega_{0}t,\;\psi_{21}\left(t\right)=\alpha_{1}\sin\omega_{0}t+\beta_{1}\cos\omega_{0}t,\\ \; & \psi_{12}\left(t\right)=\alpha_{2}\cos\omega_{0}t-\beta_{2}\sin\omega_{0}t,\;\psi_{22}\left(t\right)=\alpha_{2}\sin\omega_{0}t+\beta_{2}\cos\omega_{0}t,\\ \; & \psi_{13}\left(t\right)=\cos\omega_{0}t,\;\psi_{23}\left(t\right)=\sin\omega_{0}t,\\ \; & \psi_{14}\left(t\right)=\alpha_{4}\cos\omega_{0}t-\beta_{4}\sin\omega_{0}t,\;\psi_{24}\left(t\right)=\alpha_{4}\sin\omega_{0}t+\beta_{4}\cos\omega_{0}t. \end{array}$$

where the coefficients $\alpha_{1}$, $\beta_{1}$, $\alpha_{2}$, $\beta_{2}$, $\alpha_{4}$, $\beta_{4}$ are obtained by solving the adjoint eigenvector equation and are given explicitly in Appendix A.1 for completeness.

For $\phi_{k}\in \left(C\left[-\tau ,0\right],R^{4}\right),\;\psi_{j}\in \left(\hat{C}\left[0,\tau \right],{\left(R^{4}\right)}^{*}\right)$, the bilinear pairing can be written as:

(17) $$\langle \psi_{j}\left(t\right),\phi_{k}\left(\theta \right)\rangle =\left(\psi_{j}\left(0\right),\phi_{k}\left(0\right)\right)+\frac{f^{\prime }\left(p^{*}\right)}{\tau_{\gamma }}\int_{-\tau }^{0}\psi_{j4}\left(\xi +\tau \right)\phi_{3k}\left(\xi \right)d\xi .$$

Substituting $\Phi \left(\theta \right)$ and $\Psi \left(t\right)$ into the bilinear pairing yields the following nonsingular matrix: $J=\left(\begin{array}{cc} \psi_{11} & \psi_{12}\\ \psi_{21} & \psi_{22} \end{array}\right)$.

$$\begin{array}{cc} \psi_{11}= & \alpha_{1}-\alpha_{2}\frac{-\alpha -\beta \frac{M}{N}S^{*}}{-\beta \frac{M}{N}R^{*}}+\frac{-E_{R}\cos\left(\omega_{0}\tau \right)+E_{I}\sin\left(\omega_{0}\tau \right)}{K}\\ \; & +\alpha_{4}\frac{D_{R}}{-\beta \frac{M}{N}R^{*}\left(1-S^{*}\right)}+\frac{f^{\prime }\left(p^{*}\right)}{\tau_{\gamma }\omega_{0}K}\{\frac{1}{2}[\left(E_{I}\alpha_{4}+E_{R}\beta_{4}\right)\sin^{2}\left(\omega_{0}\tau \right)\\ \; & \;+\left(E_{I}\beta_{4}-E_{R}\alpha_{4}\right)\left(\sin\left(\omega_{0}\tau \right)\cos\left(\omega_{0}\tau \right)-\omega_{0}\tau \right)]-\frac{\omega_{0}\tau }{2}\left(E_{I}\beta_{4}+E_{R}\alpha_{4}\right)\},\\ \psi_{12}= & \alpha_{2}\frac{\omega_{0}}{-\beta \frac{M}{N}R^{*}}+\frac{-E_{R}\sin\left(\omega_{0}\tau \right)-E_{I}\cos\left(\omega_{0}\tau \right)}{K}\\ \; & +\alpha_{4}\frac{D_{I}}{-\beta \frac{M}{N}R^{*}\left(1-S^{*}\right)}+\frac{f^{\prime }\left(p^{*}\right)}{\tau_{\gamma }\omega_{0}K}\{\frac{1}{2}[\left(E_{I}\beta_{4}-E_{R}\alpha_{4}\right)\sin^{2}\left(\omega_{0}\tau \right)\\ \; & \;-\left(E_{I}\alpha_{4}+E_{R}\beta_{4}\right)\left(\sin\left(\omega_{0}\tau \right)\cos\left(\omega_{0}\tau \right)+\omega_{0}\tau \right)]\\ \; & \;+\frac{\omega_{0}\tau }{2}\left(E_{R}\beta_{4}-E_{I}\alpha_{4}\right)\},\\ \psi_{21}= & \beta_{1}-\beta_{2}\frac{-\alpha -\beta \frac{M}{N}S^{*}}{-\beta \frac{M}{N}R^{*}}+\beta_{4}\frac{D_{R}}{-\beta \frac{M}{N}R^{*}\left(1-S^{*}\right)}\\ \; & +\frac{f^{\prime }\left(p^{*}\right)}{\tau_{\gamma }\omega_{0}K}\{\frac{1}{2}[\left(E_{I}\beta_{4}-E_{R}\alpha_{4}\right)\sin^{2}\left(\omega_{0}\tau \right)\\ \; & \;+\left(E_{I}\alpha_{4}+E_{R}\beta_{4}\right)\left(\omega_{0}\tau -\sin\left(\omega_{0}\tau \right)\cos\left(\omega_{0}\tau \right)\right)]\\ \; & \;-\frac{\omega_{0}\tau }{2}\left(E_{R}\beta_{4}+E_{I}\alpha_{4}\right)\},\\ \psi_{22}= & \beta_{2}\frac{\omega_{0}}{-\beta \frac{M}{N}R^{*}}+\beta_{4}\frac{D_{I}}{-\beta \frac{M}{N}R^{*}\left(1-S^{*}\right)}\\ \; & +\frac{f^{\prime }\left(p^{*}\right)}{\tau_{\gamma }\omega_{0}K}\{-\frac{1}{2}[\left(E_{I}\alpha_{4}+E_{R}\beta_{4}\right)\sin^{2}\left(\omega_{0}\tau \right)\\ \; & \;+\left(E_{I}\beta_{4}+E_{R}\alpha_{4}\right)\left(\sin\left(\omega_{0}\tau \right)\cos\left(\omega_{0}\tau \right)+\omega_{0}\tau \right)]\\ \; & \;-\frac{\omega_{0}\tau }{2}\left(E_{I}\beta_{4}+E_{R}\alpha_{4}\right)\}. \end{array}$$

where $K=-\beta \frac{M}{N}R^{*}\left(1-S^{*}\right)\frac{f^{\prime }\left(p^{*}\right)}{\tau_{\gamma }}$.

Therefore, $\bar{\Psi }\left(t\right)=J^{-1}\Psi \left(t\right)=\left(\begin{array}{cccc} {\bar{\psi }}_{11} & {\bar{\psi }}_{12} & {\bar{\psi }}_{13} & {\bar{\psi }}_{14}\\ {\bar{\psi }}_{21} & {\bar{\psi }}_{22} & {\bar{\psi }}_{23} & {\bar{\psi }}_{24} \end{array}\right).$ where

$$\begin{array}{cc} \; & {\bar{\psi }}_{11}=k_{J}\left[\alpha_{1}\left(\psi_{22}\cos\omega_{0}t-\psi_{12}\sin\omega_{0}t\right)+\beta_{1}\left(-\psi_{22}\sin\omega_{0}t-\psi_{12}\cos\omega_{0}t\right)\right];\\ \; & {\bar{\psi }}_{12}=k_{J}\left[\alpha_{2}\left(\psi_{22}\cos\omega_{0}t-\psi_{12}\sin\omega_{0}t\right)+\beta_{2}\left(-\psi_{22}\sin\omega_{0}t-\psi_{12}\cos\omega_{0}t\right)\right];\\ \; & {\bar{\psi }}_{13}=k_{J}\left[\psi_{22}\cos\omega_{0}t-\psi_{12}\sin\omega_{0}t\right];\\ \; & {\bar{\psi }}_{14}=k_{J}\left[\alpha_{4}\left(\psi_{22}\cos\omega_{0}t-\psi_{12}\sin\omega_{0}t\right)+\beta_{4}\left(-\psi_{22}\sin\omega_{0}t-\psi_{12}\cos\omega_{0}t\right)\right];\\ \; & {\bar{\psi }}_{21}=k_{J}\left[\alpha_{1}\left(-\psi_{21}\cos\omega_{0}t+\psi_{11}\sin\omega_{0}t\right)+\beta_{1}\left(\psi_{21}\sin\omega_{0}t+\psi_{11}\cos\omega_{0}t\right)\right];\\ \; & {\bar{\psi }}_{22}=k_{J}\left[\alpha_{2}\left(-\psi_{21}\cos\omega_{0}t+\psi_{11}\sin\omega_{0}t\right)+\beta_{2}\left(\psi_{21}\sin\omega_{0}t+\psi_{11}\cos\omega_{0}t\right)\right];\\ \; & {\bar{\psi }}_{23}=k_{J}\left[-\psi_{21}\cos\omega_{0}t+\psi_{11}\sin\omega_{0}t\right];\\ \; & {\bar{\psi }}_{24}=k_{J}\left[\alpha_{4}\left(-\psi_{21}\cos\omega_{0}t+\psi_{11}\sin\omega_{0}t\right)+\beta_{4}\left(\psi_{21}\sin\omega_{0}t+\psi_{11}\cos\omega_{0}t\right)\right];\\ \; & \;k_{J}={\left(\psi_{11}\psi_{22}-\psi_{12}\psi_{21}\right)}^{-1}. \end{array}$$

When $\tau =\tau_{0}$, the characteristic equation $\Delta \left(\lambda ,\tau \right)=0$ has a pair of purely imaginary roots $\lambda_{1,2}=\pm i\omega_{0}$, while all other eigenvalues have nonzero real parts. Therefore, the phase space of a linear time-delay system can be decomposed into the sum of a center subspace and its complement, i.e., C=P⊕Q, where P is the two-dimensional center subspace spanned by the eigenvectors corresponding to the critical eigenvalues $\pm i\omega_{0}$, and Q is the complement corresponding to the remaining eigenvalues. Let $\Phi \left(\theta \right)$ be a basis for the center subspace P, and let $\bar{\Psi }\left(t\right)$ be the adjoint basis normalized by the bilinear inner product, such that $\langle \bar{\Psi },\Phi \rangle =I$. Then the dynamics of the system on the center manifold can be expressed as

(18) $$X_{t}\left(\theta \right)=\Phi \left(\theta \right)z\left(t\right)+h\left(z\left(t\right),\theta \right).$$

where $h\left(z\left(t\right),\theta \right)$ denotes the nonlinear correction on the center manifold. The delayed term is treated through the history segment $X_{t}$. For $\tau =\tau_{0}+\epsilon \tilde{\tau }$, the delayed state is evaluated as $X\left(t-\tau \right)=X_{t}\left(-\tau_{0}-\epsilon \tilde{\tau }\right)$. Using the center-mode approximation in Equation (18), we obtain

(19) $$X\left(t-\tau \right)=\Phi \left(-\tau_{0}\right)z\left(t\right)-\epsilon \tilde{\tau }\Phi^{\prime }\left(-\tau_{0}\right)z\left(t\right)+O\left({\left|z\right|}^{2}+\epsilon^{2}\left|z\right|\right).$$

Therefore, the fixed delay is retained in the reduced equation through the delay-dependent eigenfunctions, the adjoint projection, and the delay-detuning term proportional to $\epsilon \tilde{\tau }$. In this sense, the delay is not replaced by an instantaneous feedback approximation during the reduction.

In a complete Hopf normal-form calculation, the quadratic component of *h* may contribute to the cubic coefficient of the amplitude equation. In the present work, we focus on the leading-order stochastic amplitude dynamics obtained by the critical-mode projection and do not explicitly solve the homological equations for *h*. Therefore, the cubic coefficient derived below should be interpreted as a leading-order approximation rather than the full normal-form coefficient. Next, we perform a Taylor expansion of the nonlinear terms of the original system near the equilibrium point, retaining the second- and third-order nonlinear terms in the center variables; simultaneously, we retain a first-order approximation for the time-delay offset term. Under the approximation $X_{t}\left(\theta \right)=\Phi \left(\theta \right)z\left(t\right)+O\left({\left|z\right|}^{2}\right)$, we retain the direct nonlinear terms of orders $O\left({\left|z\right|}^{2}\right)$ and $O\left({\left|z\right|}^{3}\right)$, the first-order delay-detuning term $O\left(\tilde{\tau }\left|z\right|\right)$, and the Itô correction induced by the additive noise. Higher-order terms, such as $O\left({\left|z\right|}^{4}\right)$, $O\left(\tilde{\tau }{\left|z\right|}^{2}\right)$, and higher-order noise-induced corrections, are neglected. Projecting the resulting nonlinear terms, time-delay disturbance terms, and stochastic disturbance terms onto the normalized adjoint basis $\bar{\Psi }\left(t\right)$ yields the two-dimensional stochastic reduced-order equation on the center manifold:

(20) $$\begin{array}{cc} dz_{1}= & [\omega_{0}z_{2}+{\bar{\psi }}_{11}\left(0\right)F_{1}+{\bar{\psi }}_{12}\left(0\right)F_{2}+{\bar{\psi }}_{13}\left(0\right)F_{3}+{\bar{\psi }}_{14}\left(0\right)F_{4}^{\left(2\right)}+{\bar{\psi }}_{14}\left(0\right)F_{4}^{\left(3\right)}\\ \; & \;-\epsilon \tilde{\tau }\;{\bar{\psi }}_{14}\left(0\right)\frac{f^{\prime }\left(p^{*}\right)\omega_{0}}{\tau_{\gamma }K}\left(E_{I}z_{1}-E_{R}z_{2}\right)]dt+{\bar{\psi }}_{14}\left(0\right)\frac{\sigma }{\tau_{\gamma }}dW_{t},\\ dz_{2}= & [-\omega_{0}z_{1}+{\bar{\psi }}_{21}\left(0\right)F_{1}+{\bar{\psi }}_{22}\left(0\right)F_{2}+{\bar{\psi }}_{23}\left(0\right)F_{3}+{\bar{\psi }}_{24}\left(0\right)F_{4}^{\left(2\right)}+{\bar{\psi }}_{24}\left(0\right)F_{4}^{\left(3\right)}\\ \; & \;-\epsilon \tilde{\tau }\;{\bar{\psi }}_{24}\left(0\right)\frac{f^{\prime }\left(p^{*}\right)\omega_{0}}{\tau_{\gamma }K}\left(E_{I}z_{1}-E_{R}z_{2}\right)]dt+{\bar{\psi }}_{24}\left(0\right)\frac{\sigma }{\tau_{\gamma }}dW_{t}. \end{array}$$

where

$$F_{1}=-\beta \frac{M}{N}\left(-\frac{-\alpha -\beta \frac{M}{N}S^{*}}{-\beta \frac{M}{N}R^{*}}z_{1}^{2}+\frac{\omega_{0}}{-\beta \frac{M}{N}R^{*}}z_{1}z_{2}\right);$$

$$\begin{array}{cc} \; & F_{2}=-\frac{\left(\left(\alpha +\beta \frac{M}{N}S^{*}\right)D_{R}z_{1}^{2}+\left(\omega_{0}D_{R}-\left(-\alpha -\beta \frac{M}{N}S^{*}\right)D_{I}\right)z_{1}z_{2}+\omega_{0}D_{I}z_{2}^{2}\right)}{{\left(-\beta \frac{M}{N}R^{*}\right)}^{2}\left(1-S^{*}\right)}\\ \; & \;\;+\beta \frac{M}{N}\left(-\frac{-\alpha -\beta \frac{M}{N}S^{*}}{-\beta \frac{M}{N}R^{*}}z_{1}^{2}+\frac{\omega_{0}}{-\beta \frac{M}{N}R^{*}}z_{1}z_{2}\right); \end{array}$$

$$\begin{array}{cc} \; & F_{3}=\frac{\nu M\left(\left(\alpha +\beta \frac{M}{N}S^{*}\right)D_{R}z_{1}^{2}+\left(\omega_{0}D_{R}-\left(-\alpha -\beta \frac{M}{N}S^{*}\right)D_{I}\right)z_{1}z_{2}+\omega_{0}D_{I}z_{2}^{2}\right)}{{\left(-\beta \frac{M}{N}R^{*}\right)}^{2}\left(1-S^{*}\right)};\\ \; & \;\;-\nu \beta^{2}M\left(-\frac{-\alpha -\beta \frac{M}{N}S^{*}}{-\beta \frac{M}{N}R^{*}}z_{1}^{2}+\frac{\omega_{0}}{-\beta \frac{M}{N}R^{*}}z_{1}z_{2}\right);\\ \; & F_{4}^{\left(2\right)}=\frac{f^{\prime \prime }\left(p^{*}\right)}{2\tau_{\gamma }K^{2}}{\left(E_{R}z_{1}+E_{I}z_{2}\right)}^{2};\\ \; & F_{4}^{\left(3\right)}=\frac{f^{\prime \prime \prime }\left(p^{*}\right)}{6\tau_{\gamma }K^{3}}{\left(E_{R}z_{1}+E_{I}z_{2}\right)}^{3}. \end{array}$$

### 3.2. Stochastic Averaging and Reduced Amplitude Equation

The polar coordinate transformation is performed using the stochastic averaging method, as follows:

(21) $$\left\{\begin{array}{c} z_{1}\left(t\right)=\rho \left(t\right)\cos\theta \\ z_{2}\left(t\right)=-\rho \left(t\right)\sin\theta \\ \theta =\omega_{0}t+\varphi \left(t\right) \end{array}\right..$$

ρ(t) and $\varphi \left(t\right)$ represent the amplitude and phase of the solution, respectively. We can obtain stochastic differential equations for the amplitude process ρ(t) and the phase process $\varphi \left(t\right)$. Let

$$\begin{array}{cc} P_{1} & ={\bar{\psi }}_{14}\left(0\right)\frac{\sigma }{\tau_{\gamma }},\\ P_{2} & ={\bar{\psi }}_{24}\left(0\right)\frac{\sigma }{\tau_{\gamma }},\\ Q_{1}\left(\rho ,\theta \right) & ={\bar{\psi }}_{11}\left(0\right)F_{1}\left(\rho ,\theta \right)+{\bar{\psi }}_{12}\left(0\right)F_{2}\left(\rho ,\theta \right)+{\bar{\psi }}_{13}\left(0\right)F_{3}\left(\rho ,\theta \right)\\ \; & \;+{\bar{\psi }}_{14}\left(0\right)\left(F_{4}^{\left(2\right)}\left(\rho ,\theta \right)+F_{4}^{\left(3\right)}\left(\rho ,\theta \right)\right)-\epsilon \tilde{\tau }{\bar{\psi }}_{14}\left(0\right)\frac{f^{\prime }\left(p^{*}\right)\omega_{0}}{\tau_{\gamma }K}\rho \left(E_{I}\cos\theta +E_{R}\sin\theta \right),\\ Q_{2}\left(\rho ,\theta \right) & ={\bar{\psi }}_{21}\left(0\right)F_{1}\left(\rho ,\theta \right)+{\bar{\psi }}_{22}\left(0\right)F_{2}\left(\rho ,\theta \right)+{\bar{\psi }}_{23}\left(0\right)F_{3}\left(\rho ,\theta \right)\\ \; & \;+{\bar{\psi }}_{24}\left(0\right)\left(F_{4}^{\left(2\right)}\left(\rho ,\theta \right)+F_{4}^{\left(3\right)}\left(\rho ,\theta \right)\right)-\epsilon \tilde{\tau }{\bar{\psi }}_{24}\left(0\right)\frac{f^{\prime }\left(p^{*}\right)\omega_{0}}{\tau_{\gamma }K}\rho \left(E_{I}\cos\theta +E_{R}\sin\theta \right). \end{array}$$

The stochastic differential equation is:

(22) $$\begin{array}{cc} \; & d\rho =\left[Q_{1}\left(\rho ,\theta \right)\cos\theta -Q_{2}\left(\rho ,\theta \right)\sin\theta +\frac{{\left(P_{2}\cos\theta +P_{1}\sin\theta \right)}^{2}}{2\rho }\right]dt\\ \; & \;\;+\left(P_{1}\cos\theta -P_{2}\sin\theta \right)dW_{t},\\ \; & d\varphi =\left[-\frac{Q_{1}\left(\rho ,\theta \right)\sin\theta +Q_{2}\left(\rho ,\theta \right)\cos\theta }{\rho }+\frac{\left(P_{1}\sin\theta +P_{2}\cos\theta \right)\left(P_{1}\cos\theta -P_{2}\sin\theta \right)}{\rho^{2}}\right]dt\\ \; & \;\;-\frac{P_{1}\sin\theta +P_{2}\cos\theta }{\rho }dW_{t}. \end{array}$$

where

$$\begin{array}{cc} F_{1}\left(\rho ,\theta \right)= & \beta \frac{M}{N}\frac{\rho^{2}}{-\beta \frac{M}{N}R^{*}}\left[\left(-\alpha -\beta \frac{M}{N}S^{*}\right)\cos^{2}\theta +\omega_{0}\cos\theta \sin\theta \right],\\ F_{2}\left(\rho ,\theta \right)= & \frac{\rho^{2}}{{\left(-\beta \frac{M}{N}R^{*}\right)}^{2}\left(1-S^{*}\right)}[\left(-\alpha -\beta \frac{M}{N}S^{*}\right)D_{R}\cos^{2}\theta \\ \; & \;+\left(\omega_{0}D_{R}-\left(-\alpha -\beta \frac{M}{N}S^{*}\right)D_{I}\right)\cos\theta \sin\theta -\omega_{0}D_{I}\sin^{2}\theta ]\\ \; & \;-\frac{N^{2}}{M^{2}R^{*}}\rho^{2}\left[\left(-\alpha -\beta \frac{M}{N}S^{*}\right)\cos^{2}\theta +\omega_{0}\cos\theta \sin\theta \right],\\ F_{3}\left(\rho ,\theta \right)= & \nu M\frac{\rho^{2}}{{\left(-\beta \frac{M}{N}R^{*}\right)}^{2}\left(1-S^{*}\right)}[\left(-\alpha -\beta \frac{M}{N}S^{*}\right)D_{R}\cos^{2}\theta \\ \; & \;+\left(\omega_{0}D_{R}-\left(-\alpha -\beta \frac{M}{N}S^{*}\right)D_{I}\right)\cos\theta \sin\theta -\omega_{0}D_{I}\sin^{2}\theta ]\\ \; & \;+\nu N^{2}\frac{\rho^{2}}{MR^{*}}\left[\left(-\alpha -\beta \frac{M}{N}S^{*}\right)\cos^{2}\theta +\omega_{0}\cos\theta \sin\theta \right],\\ F_{4}^{\left(2\right)}\left(\rho ,\theta \right)= & \frac{f^{\prime \prime }\left(p^{*}\right)}{2\tau_{\gamma }K^{2}}\rho^{2}{\left(E_{R}\cos\theta -E_{I}\sin\theta \right)}^{2},\\ F_{4}^{\left(3\right)}\left(\rho ,\theta \right)= & \frac{f^{\prime \prime \prime }\left(p^{*}\right)}{6\tau_{\gamma }K^{3}}\rho^{3}{\left(E_{R}\cos\theta -E_{I}\sin\theta \right)}^{3}. \end{array}$$

Since both ρ(t) and $\varphi \left(t\right)$ vary slowly compared with the fast angular variable, the stochastic averaging method can be applied to average the periodic term containing θ over the fast period $\frac{2\pi }{\omega_{0}}$. After averaging, the amplitude process ρ(t) can be approximated as a one-dimensional Markovian diffusion process, and the corresponding Itô stochastic differential equation is

(23) $$d\rho =\left(a\rho +b\rho^{3}+\frac{D}{2\rho }\right)dt+cdB_{t}.$$

where

$$\begin{array}{cc} \; & a=\frac{\epsilon \tilde{\tau }}{2}\frac{f^{\prime }\left(p^{*}\right)\omega_{0}}{\tau_{\gamma }K}\left(-{\bar{\psi }}_{14}\left(0\right)E_{I}+{\bar{\psi }}_{24}\left(0\right)E_{R}\right),\\ \; & b=\frac{f^{\prime \prime \prime }\left(p^{*}\right)}{16\tau_{\gamma }K^{3}}\left({\bar{\psi }}_{14}\left(0\right)E_{R}+{\bar{\psi }}_{24}\left(0\right)E_{I}\right)\left(E_{R}^{2}+E_{I}^{2}\right),\\ \; & c=\frac{\sigma }{\tau_{\gamma }}\sqrt{\frac{{\bar{\psi }}_{14}^{2}\left(0\right)+{\bar{\psi }}_{24}^{2}\left(0\right)}{2}},\\ \; & D=\frac{\sigma^{2}}{2\tau_{\gamma }^{2}}\left({\bar{\psi }}_{14}^{2}\left(0\right)+{\bar{\psi }}_{24}^{2}\left(0\right)\right). \end{array}$$

Here, *b* denotes the cubic coefficient obtained from the present leading-order center-mode projection and stochastic averaging. Since the quadratic correction h(z,θ) in the center-manifold representation is not explicitly solved, possible additional cubic contributions induced by *h* are not included in this coefficient. Therefore, the amplitude equation is interpreted as a leading-order near-Hopf approximation rather than the full normal-form amplitude equation. Moreover, by the definitions of *c* and *D*, we have $D=c^{2}$. Since $\rho \left(t\right)$ represents an amplitude variable, the diffusion process is considered on the half-line ρ≥0. The singular drift term $\frac{D}{2\rho }$ is the Itô correction induced by the polar coordinate transformation.

It should be emphasized that Equation (23) is valid only in the local, near-Hopf, weak-noise regime. Specifically, at $\tau =\tau_{0}$, assume that the deterministically linearized time-delay system has a pair of simple critical roots $\pm i\omega_{0}$, while the remaining eigenroots lie far from the imaginary axis. Assume that the delay detuning $\tau -\tau_{0}$, the central mode amplitude, and the noise intensity are all sufficiently small such that the rate of change of the amplitude is very slow relative to the phase of the fast oscillation with period $2\pi /\omega_{0}$. Under these conditions of time-scale separation, the phase-dependent term in Equation (22) can be averaged over one oscillation period. Since the random perturbation is modeled as white Brownian noise, its correlation time in the idealized model is zero. Therefore, within this local near-Hopf weak-noise regime, the fixed response delay does not invalidate the stochastic averaging procedure, because its effect is retained through the delay-dependent eigenfunctions, the adjoint projection, and the delay-detuning term. However, this approximation does not hold for large delay detuning, strong noise, or cases where other characteristic roots are close to the imaginary axis.

In this paper, the delay-induced Hopf bifurcation refers to the deterministic critical transition at $\tau =\tau_{0}$, whereas the stochastic P-bifurcation refers to the qualitative change in the stationary probability density of the amplitude process under stochastic perturbations. Since this paper introduces additive Gaussian white noise, the zero-amplitude state no longer remains a strictly invariant solution under stochastic perturbations. Therefore, this paper primarily discusses stochastic P-bifurcations [36] from the perspective of changes in the structure of the steady-state probability density. The Fokker–Planck equation associated with Equation (23) can be written as

$$\frac{\partial p\left(\rho ,t\right)}{\partial t}=-\frac{\partial J\left(\rho ,t\right)}{\partial \rho }.$$

where the probability current is

$$J\left(\rho ,t\right)=\left(a\rho +b\rho^{3}+\frac{D}{2\rho }\right)p\left(\rho ,t\right)-\frac{D}{2}\frac{\partial p\left(\rho ,t\right)}{\partial \rho }.$$

Because the amplitude process is defined on $\left[0,\infty \right)$, we impose the zero-flux boundary condition $J\left(0,t\right)=0$, $J\left(\infty ,t\right)=0$. Equivalently, ρ=0 is treated as a reflecting boundary for the amplitude process. Under the stationary condition $J\left(\rho \right)=0$, we obtain

$$\frac{dp_{st}\left(\rho \right)}{d\rho }=\frac{2}{D}\left(a\rho +b\rho^{3}+\frac{D}{2\rho }\right)p_{st}\left(\rho \right).$$

Therefore,

(24) $$p_{st}\left(\rho \right)=\frac{1}{Z_{\rho }}\rho \;\exp\;\left(\frac{a}{D}\rho^{2}+\frac{b}{2D}\rho^{4}\right),\;\rho \geq 0$$

where $Z_{\rho }$ is a normalization constant.

### 3.3. Stationary Density and Stochastic P-Bifurcation

To more clearly illustrate the change in the location of the probability density peak, let $Y=\rho^{2}$. By Itô’s formula, we obtain

(25) $$dY=\left(2aY+2bY^{2}+2D\right)dt+2\sqrt{DY}dB_{t},\;Y\geq 0$$

The corresponding steady-state probability density is

(26) $$p_{st}\left(y\right)=\frac{1}{Z_{Y}}\exp\left(\frac{a}{D}y+\frac{b}{2D}y^{2}\right),\;y\geq 0$$

Here, $Z_{\rho }$ and $Z_{Y}$ represent the normalization constants for the steady-state probability density functions of ρ and $Y=\rho^{2}$, respectively. They are obtained from the requirement that the integral of the corresponding probability density over the half-line equals 1. Since the variable transformation $Y=\rho^{2}$ satisfies dy=2ρdρ, the two constants satisfy $Z_{Y}=2Z_{\rho }$. In the subsequent entropy analysis, we use $Z_{Y}$ because entropy is defined based on the steady-state density of *Y*.

When b<0, as y→+∞, we have $\frac{a}{D}y+\frac{b}{2D}y^{2}\to -\infty$, so the steady-state probability density can be normalized. Taking the logarithm of $p_{st}\left(y\right)$ and differentiating yields:

(27) $$\frac{d\ln p_{st}\left(y\right)}{dy}=\frac{a}{D}+\frac{b}{D}y.$$

**Figure 2:**
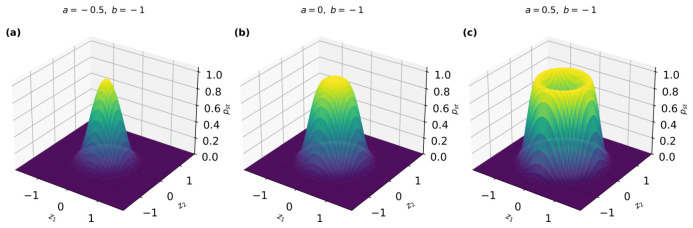
Stochastic P-bifurcation diagram of the system for different values of *a*: (**a**) a=−0.5,b=−1. (**b**) a=0,b=−1. (**c**) a=0.5,b=−1.

Setting this derivative equal to zero yields the stationary point $y^{*}=-\frac{a}{b}$. Under the condition b<0, since y≥0, there are no interior stationary points when a<0, and the mode of the stationary density lies on the boundary y=0; when a=0, the system is in a critical state; when a>0, the mode of the stationary density shifts to the interior point $y^{*}=-\frac{a}{b}$. Therefore, the system undergoes a stochastic P-bifurcation at a=0. The corresponding evolution of the probability density is shown in Figure 2.

When b=0, the steady-state probability density reduces to the linear form, in which case the steady-state probability density function is

(28) $$p_{st}\left(y\right)=\frac{1}{Z}\exp\left(\frac{a}{D}y\right).$$

In this case, a stationary probability density exists only if a<0; if a≥0, the distribution is non-normalizable, and there is no stationary probability density function.

When b>0, $\frac{a}{D}y+\frac{b}{2D}y^{2}\to +\infty$, y→+∞; therefore, the steady-state probability density cannot be normalized. Consequently, the system does not have a stationary probability density function either.

### 3.4. Entropy-Based Uncertainty Characterization

Although stochastic P-bifurcation reveals qualitative changes in the steady-state probability density, it does not fully quantify how stochastic perturbations redistribute probability mass in amplitude space. To characterize the global expansion of uncertainty and changes in probability structure caused jointly by time delays and noise, this paper introduces an information measure based on entropy.

The reason for using Shannon entropy rather than variance or kurtosis is that the present study focuses on the reorganization of the entire stationary probability density. For the stationary amplitude-squared variable *Y*, variance and kurtosis are finite-order moment descriptors, for example,

$$\mathrm{Var}\left(Y\right)=E\left[Y^{2}\right]-{\left(E\left[Y\right]\right)}^{2},$$

and

$$\mathrm{Kurt}\left(Y\right)=\frac{E[{\left(Y-E\left[Y\right]\right)}^{4}]}{{\left(\mathrm{Var}(Y)\right)}^{2}}.$$

These quantities characterize dispersion and tail weight, but they do not uniquely determine the underlying probability density. Therefore, they may fail to distinguish stationary densities with similar moments but different modal structures, boundary concentrations, or probability-mass redistributions. In contrast, Shannon entropy is defined as a functional of the full probability density and therefore provides a direct scalar measure of the overall uncertainty associated with the stationary amplitude distribution. This makes entropy more suitable for characterizing probabilistic structural reorganization in the present stochastic P-bifurcation analysis.

For the case where b<0, the steady-state probability density given by Equation (26) is normalizable. Since the stochastic P-bifurcation primarily manifests as a qualitative change in the steady-state probability density of the amplitude-squared variable $Y=\rho^{2}$, we define Shannon entropy as follows:

(29) $$H_{Y}=-\int_{0}^{\infty }p_{st}\left(y\right)\ln p_{st}\left(y\right)dy.$$

where $p_{st}\left(y\right)$ denotes the steady-state probability density of the amplitude-squared process $Y=\rho^{2}$. Substituting Equation (26) into the above definition of entropy yields

(30) $$H_{Y}=\ln Z_{Y}-\frac{a}{D}E\left[Y\right]-\frac{b}{2D}E\left[Y^{2}\right].$$

This expression shows that, for the specific stationary density in Equation (26), the entropy of the steady-state amplitude distribution can be expressed through the normalization factor $Z_{Y}$ and the first two raw moments of *Y*. Nevertheless, $H_{Y}$ should not be interpreted as a moment descriptor such as variance or kurtosis, because it is derived from the logarithmic functional definition of Shannon entropy applied to the complete stationary density on y≥0. Thus, $H_{Y}$ summarizes the overall uncertainty associated with the stationary amplitude distribution, including distributional spreading and probability-mass redistribution.

Accordingly, this entropy measure does not redefine the stochastic P-bifurcation threshold. Instead, it characterizes the information-theoretic consequence of the transition by quantifying the spreading of the steady-state amplitude distribution. Specifically, the parameter *a* captures the influence of response delay deviations relative to the Hopf threshold, while *D* is proportional to the noise intensity. Therefore, $H_{Y}$ can serve as a scalar metric to quantify how response delay and stochastic perturbations jointly alter the uncertainty in the amplitude of foraging oscillations in ant colonies.

When the probability mass is concentrated primarily around Y=0, the entropy value is relatively low, indicating that the amplitudes of the system’s random oscillations are relatively concentrated; however, following a stochastic P-bifurcation, the steady-state probability density expands into the region of non-zero amplitudes, causing the probability distribution to become more dispersed and thereby increasing the uncertainty of the distribution.

## 4. Numerical Simulation and Analysis

To further validate the theoretical analysis results presented earlier regarding system stability, Hopf bifurcations, and stochastic P-bifurcations, and to elucidate the effects of response delays and stochastic perturbations on the foraging dynamics of ants, this section primarily uses the parameter values listed in Table 1 and employs the Euler–Maruyama method to perform numerical simulations of the established pheromone feedback foraging model, which incorporates time delays and noise. It should be emphasized that all variables and parameters used in the numerical simulation are dimensionless (detailed numerical settings, including the time step, total simulation time, initial history functions, delay interpolation method, transient removal, Monte Carlo sample size, and density estimation procedure, are provided in Appendix B, Table A1).

**Figure 3:**
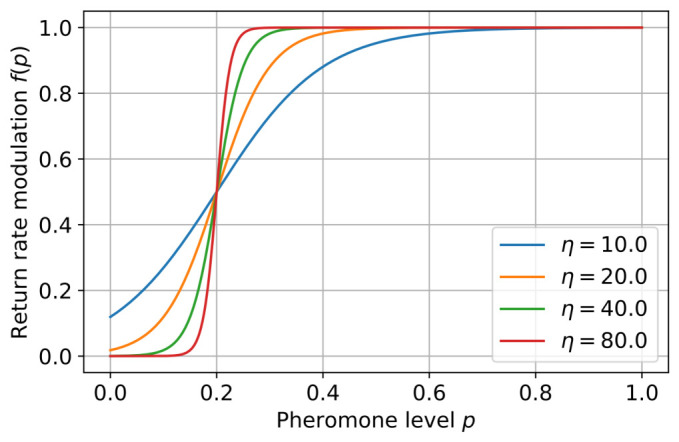
Variation of $f\left(p\right)$ with pheromone concentration *p* under different parameters η.

First, to characterize the relationship between pheromone concentration and return-to-nest rate, we consider the logistic function $f\left(p\right)=\frac{\kappa }{1+\exp\left(-\eta \left(p-p_{0}\right)\right)}$, with κ=1 and $p_{0}=0.2$ fixed. Figure 3 shows $f\left(p\right)$ for different values of η.

To better highlight the threshold effect and strong nonlinear response characteristics in pheromone feedback, and to represent a high-sensitivity pheromone response, we set η=80 as the parameter for the numerical simulation.

Using the parameter values in Table 1 and solving the equilibrium Equation (2), we obtain $E^{*}=\left(R^{*},S^{*},p^{*},\gamma^{*}\right)$, where $R^{*}=0.159511$, $S^{*}=0.878191$, $p^{*}=0.189110$, $\gamma^{*}=0.345004$. To make the numerical verification of the Hopf threshold more transparent, the key computational results are summarized in Table 2. The detailed calculation procedure is provided in Appendix A.2.

As shown in Table 2, L(0.2)<0, and therefore H(1) is satisfied. Moreover, I≠0 and Re(dλ/dτ)τ=τ0≠0, indicating that H(2) and the transversality condition hold. Therefore, the first Hopf bifurcation threshold is $\tau_{0}=7.421015$. According to the dimensional interpretation introduced in Section 2, this dimensionless Hopf threshold can be converted into a physical critical delay as $T_{\mathrm{delay}}^{\left(0\right)}=\tau_{0}T_{\gamma }=7.421015T_{\gamma }$. Therefore, once the characteristic response time $T_{\gamma }$ is specified for a given ant species or experimental condition, the model delay can be directly expressed in seconds or minutes. For example, if $T_{\gamma }=30$ s, the corresponding dimensional critical delay is approximately 3.71 min. This conversion provides a dimensional calibration basis for interpreting the effective delay parameter, while leaving the nondimensional Hopf threshold and bifurcation structure unchanged. For the parameter values used in this section, the cubic coefficient in the reduced amplitude equation satisfies b<0, which ensures the normalizability of the stationary density in Equation (26).

### 4.1. Deterministic Hopf Validation

**Figure 4:**
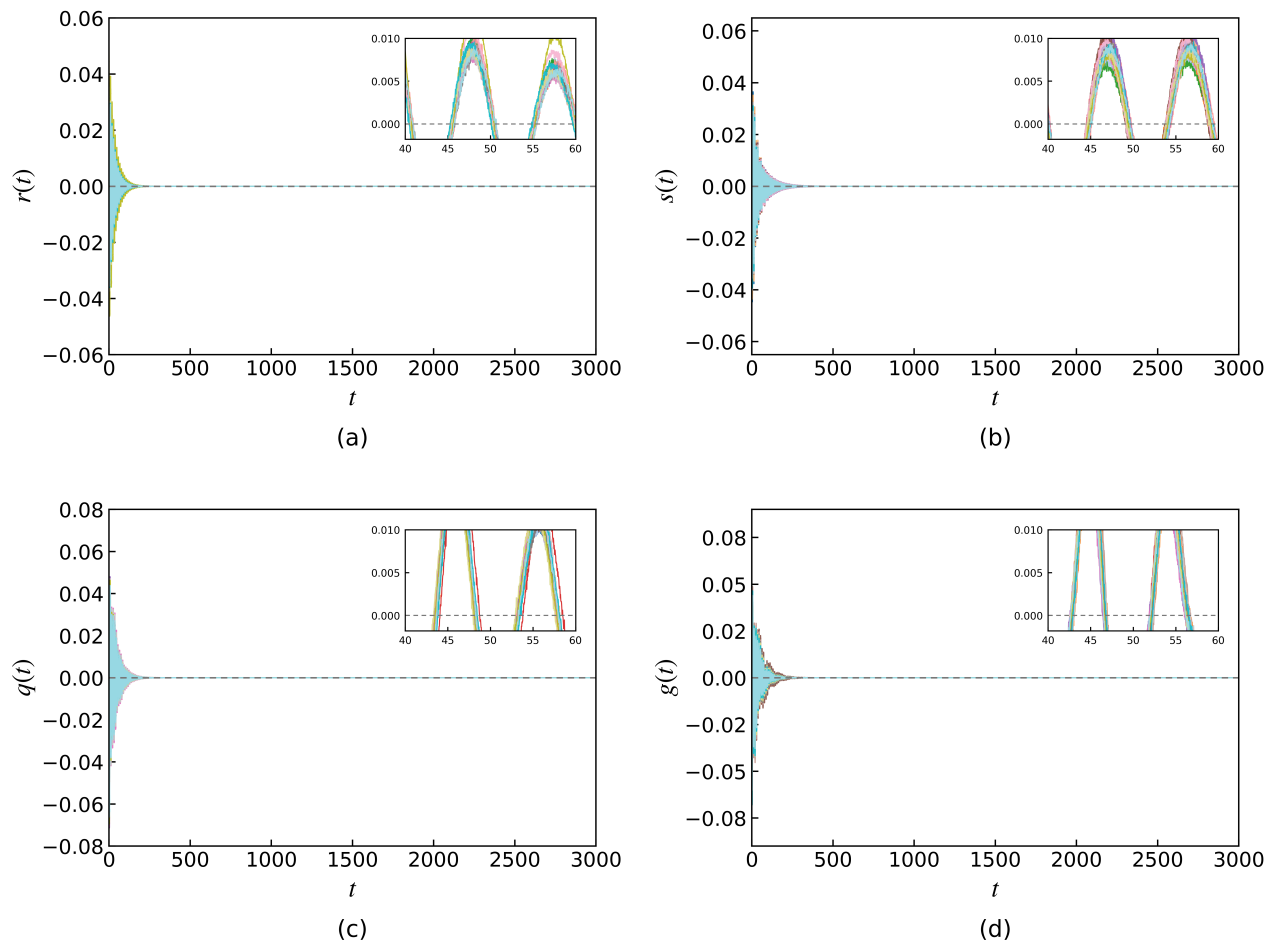
Time series of *r*, *s*, *q*, *g* for the parameter $\tau =7.2<\tau_{0}$: (**a**) $r\left(t\right)$. (**b**) $s\left(t\right)$. (**c**) $q\left(t\right)$. (**d**) $g\left(t\right)$.

**Figure 5:**
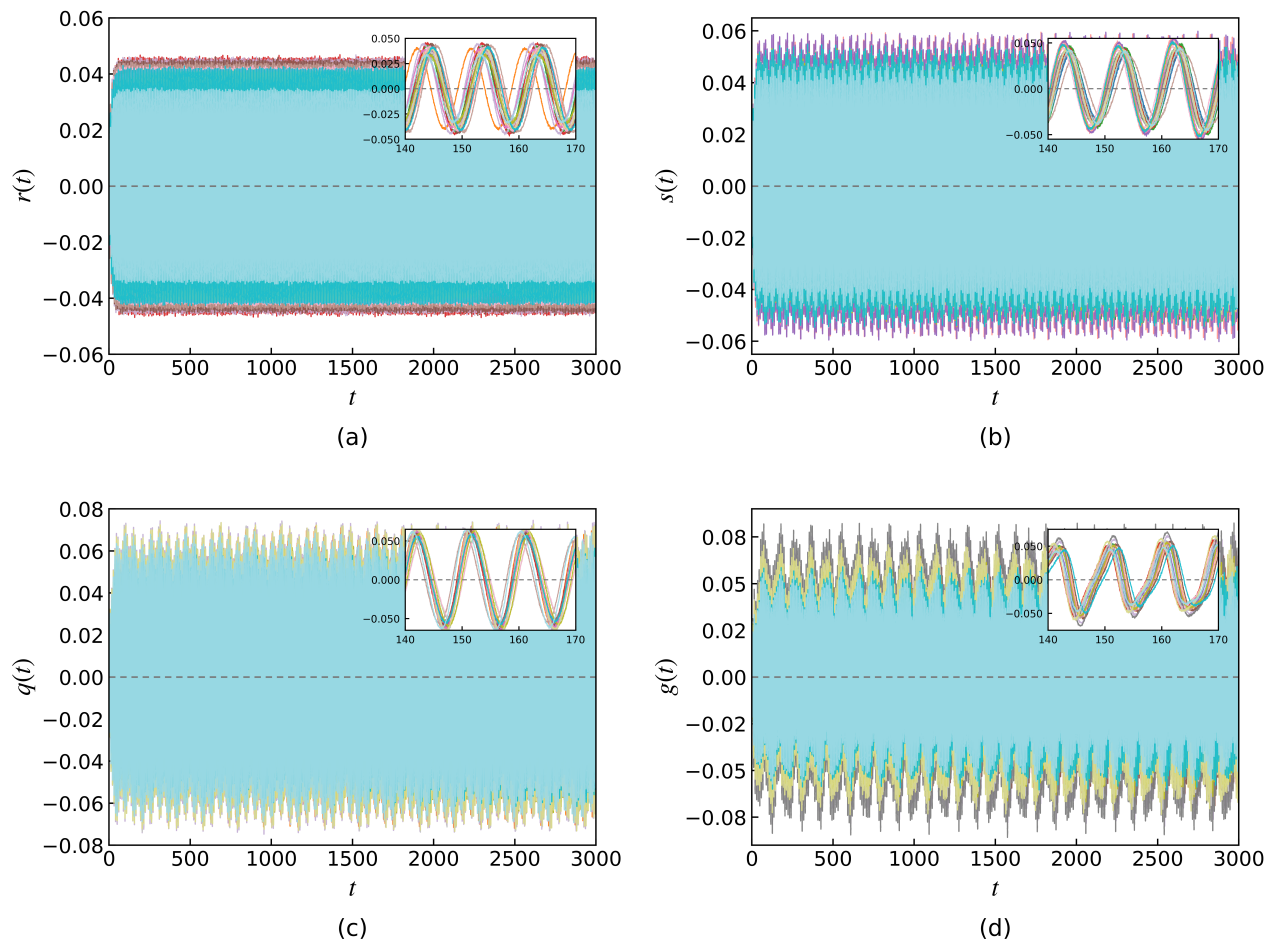
Time series of *r*, *s*, *q*, *g* for the parameter $\tau =7.6>\tau_{0}$: (**a**) $r\left(t\right)$. (**b**) $s\left(t\right)$. (**c**) $q\left(t\right)$. (**d**) $g\left(t\right)$.

To verify the theoretical results discussed earlier regarding time-delay-induced Hopf bifurcations, we first disregard the effects of stochastic perturbations, set the noise intensity to zero, and select τ=7.2 and τ=7.6. We simulate the deterministic system from several small perturbations around $E^{*}$ to examine local stability and then plot the time series of the system state variables $r\left(t\right),s\left(t\right),q\left(t\right),g\left(t\right)$, as shown in Figure 4 and Figure 5. When $\tau =7.2<\tau_{0}$, all state variables exhibit damped oscillations and eventually converge to a steady state, indicating that the system’s equilibrium point remains stable at this time; when $\tau =7.6>\tau_{0}$, the oscillation amplitudes of the variables no longer decay but instead form sustained periodic oscillations, indicating that a Hopf bifurcation occurs at the critical delay. This suggests that during the foraging process, if the response time of the return-to-nest rate to changes in pheromone concentration remains within the critical threshold, a stable coordination can gradually be achieved among foraging ants, supplier ants, receiver ants, and pheromone feedback. However, once the response delay exceeds this threshold, the lag effect between pheromone feedback and behavioral transitions disrupts the original equilibrium, causing sustained oscillations in ant movement in and out of the nest and in pheromone concentration.

### 4.2. Stochastic Time-Series Response

**Figure 6:**
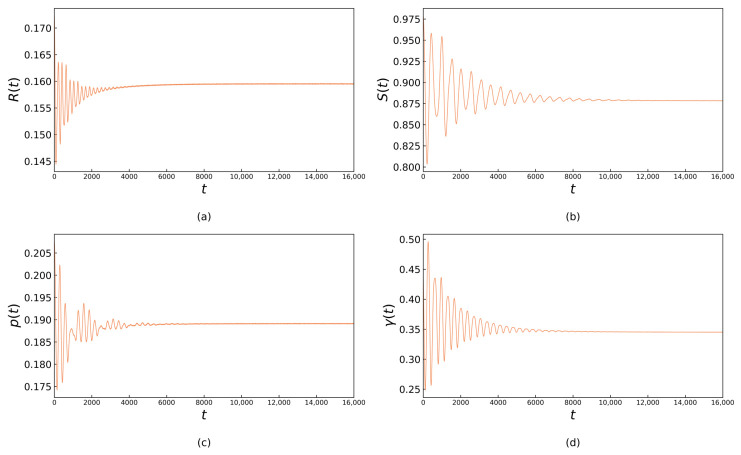
Time series of $R\left(t\right),\;S\left(t\right),\;p\left(t\right),\;\gamma \left(t\right)$ under Gaussian white noise with σ=0.02 for $\tau =7.30<\tau_{0}$: (**a**) $R\left(t\right)$. (**b**) $S\left(t\right)$. (**c**) $p\left(t\right)$. (**d**) $\gamma \left(t\right)$.

**Figure 7:**
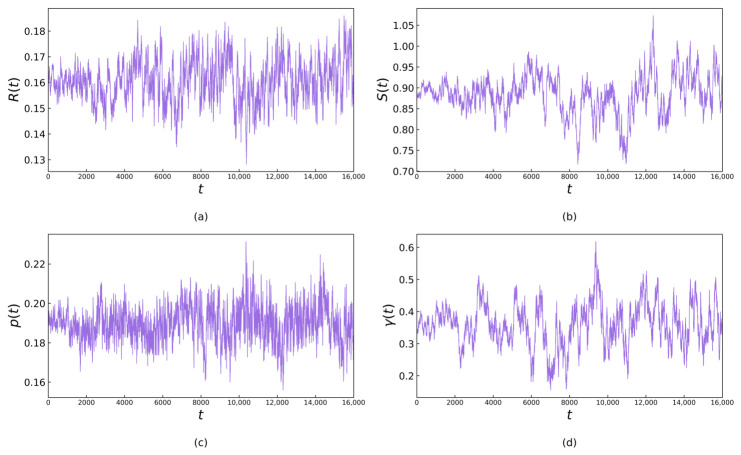
Time series of $R\left(t\right),\;S\left(t\right),\;p\left(t\right),\;\gamma \left(t\right)$ under Gaussian white noise with σ=0.02 for $\tau =7.55>\tau_{0}$: (**a**) $R\left(t\right)$. (**b**) $S\left(t\right)$. (**c**) $p\left(t\right)$. (**d**) $\gamma \left(t\right)$.

To further verify the system’s dynamic behavior near the critical delay under stochastic perturbations, we consider Gaussian white noise with noise intensity σ=0.02. We select $\tau =7.30<\tau_{0}$ and $\tau =7.55>\tau_{0}$, respectively, and plot the time series graphs of $R\left(t\right),S\left(t\right),p\left(t\right),\gamma \left(t\right)$ (see Figure 6 and Figure 7). As can be seen from the figures, under noise perturbation, when $\tau =7.30<\tau_{0}$, the deterministic oscillatory component is damped, and the stochastic trajectory remains confined in a small neighborhood of the coexistence equilibrium, indicating that the system still possesses a certain degree of disturbance resistance at this point; however, when $\tau =7.55>\tau_{0}$, all variables exhibit continuous and irregular random fluctuations. This indicates that once the response delay exceeds the critical value, noise amplifies the lag effect between pheromone feedback and ant behavioral transitions, and the stationary distribution shifts from a neighborhood of the equilibrium to a nonzero-amplitude oscillatory region. This demonstrates that response delay and stochastic perturbations jointly influence the stability of the ant foraging system and also supports the validity of the stochastic reduction and bifurcation analysis.

### 4.3. Stationary Density and Most Probable Amplitude

**Figure 8:**
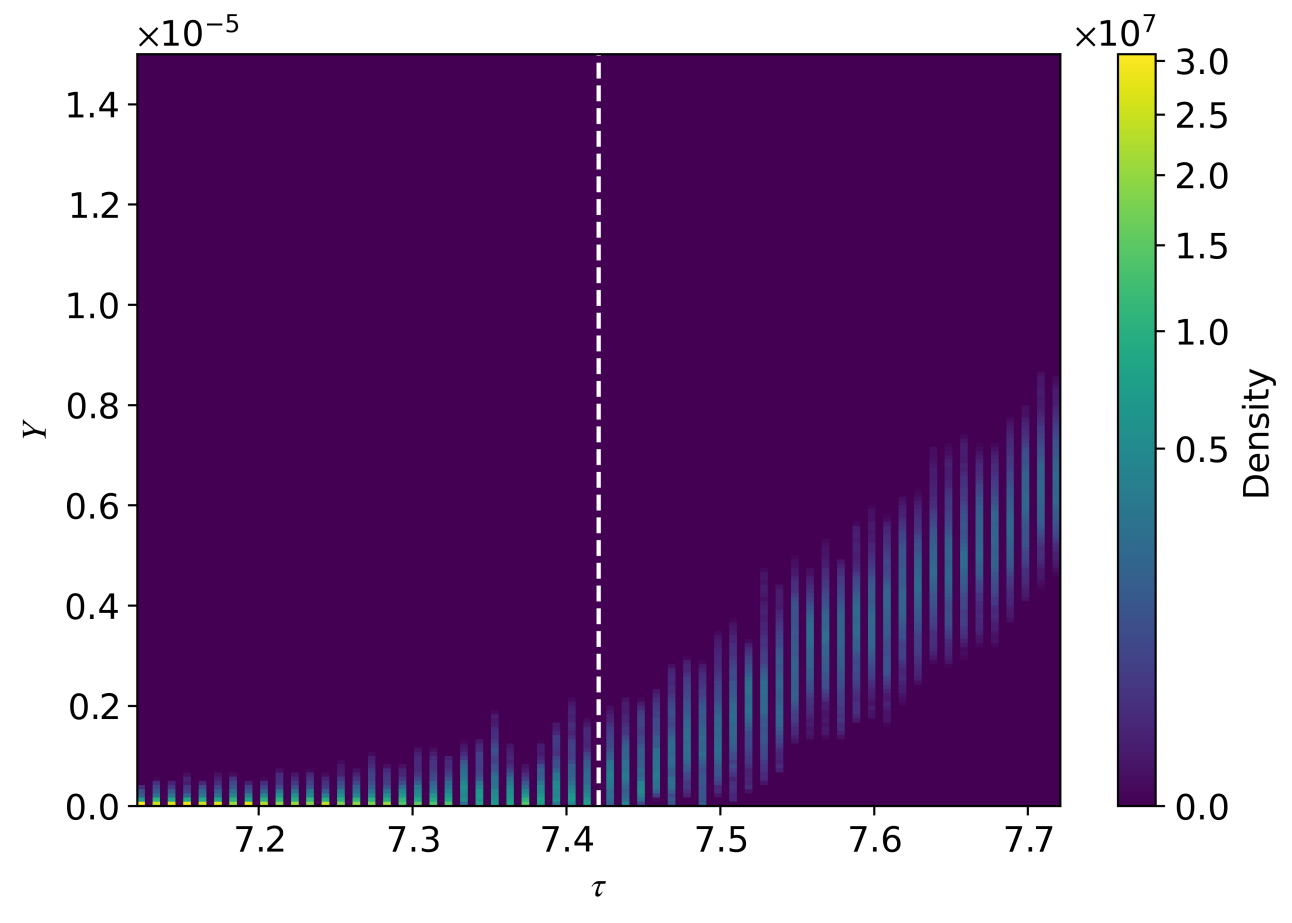
Evolution of the probability density of the amplitude variable *Y* with respect to the response time delay τ. The white dashed line denotes the critical delay $\tau_{0}$.

Since the primary dynamics of the original system near the Hopf critical point are determined by the center mode corresponding to the critical eigenvalue, the amplitude variable Y directly reflects changes in the system’s oscillation intensity. Compared to the variables of the original system, the amplitude equation not only reduces the system’s dimensionality but also facilitates the determination of whether a stochastic P-bifurcation occurs from the perspectives of steady-state probability density and most probable value. Therefore, we proceed to conduct numerical simulations focusing on the structure of the steady-state probability density of the amplitude variable *Y*. Figure 8 illustrates the evolution of the probability density on the $\left(\tau ,Y\right)$ plane, providing a visual representation of the overall trend of stochastic P-bifurcation; the white dashed line represents the critical delay $\tau_{0}$. When $\tau <\tau_{0}$, the probability density is primarily concentrated near Y=0; once $\tau >\tau_{0}$, the peak of the probability density gradually moves away from zero and shifts toward the positive region, indicating that the system transitions from a state of small perturbations near zero amplitude to a state of oscillation with non-zero amplitude, suggesting that the system undergoes a stochastic P-bifurcation near $\tau =\tau_{0}$.

**Figure 9:**
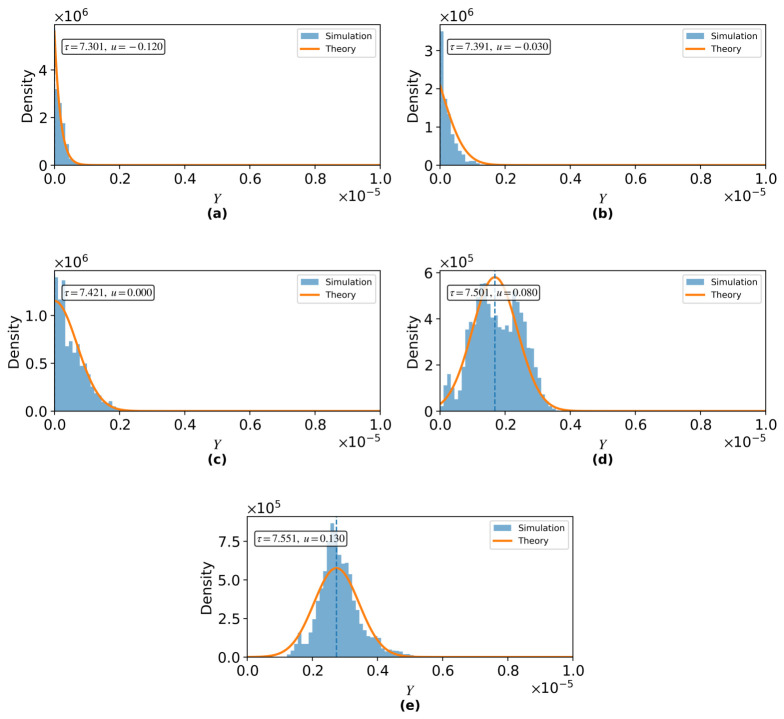
Steady-state probability density distribution of the amplitude variable *Y* under different response time delays: (**a**) τ=7.301, u=−0.120. (**b**) τ=7.391, u=−0.030. (**c**) τ=7.421, u=0.000. (**d**) τ=7.501, u=0.080. (**e**) τ=7.551, u=0.130.

By selecting several values of τ on either side of the critical delay and letting $u=\tau -\tau_{0}$ denote the offset of the actual delay relative to the critical delay, we compare the numerical simulation histogram with the theoretical steady-state probability density curve (see Figure 9). The simulated histograms are obtained from numerical simulations of the reduced amplitude SDE for *Y*, while the theoretical curves are computed from the stationary density Formula (26). We find that when u<0, the peak of the probability density lies near Y=0; when u=0, the system is in a critical state; when u>0, the peak of the probability density shifts to a positive interior point and gradually moves to the right as τ increases. This verifies the validity of the theoretical probability density function and the P-bifurcation criterion.

Next, we define the most probable value of the amplitude variable *Y*

(31) $$Y_{mp}=\arg\max_{y\geq 0}p_{st}\left(y\right).$$

Using the steady-state probability density expression obtained in Section 3, we obtain

(32) $$Y_{mp}=\max\left(-\frac{a\left(\tau \right)}{b},0\right).$$

**Figure 10:**
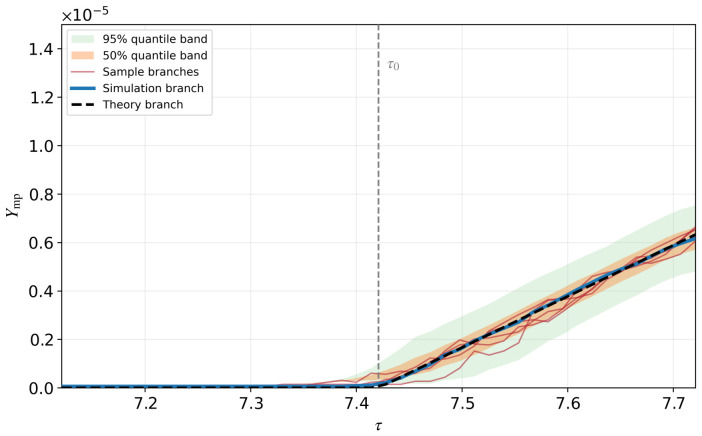
Stochastic P-bifurcation diagram of the most probable value $Y_{mp}$ of the amplitude variable *Y* versus response delay τ.

We extract the most probable value $Y_{mp}$ of the amplitude variable *Y* for different response delays, plot a stochastic P-bifurcation diagram of $Y_{mp}$ as a function of τ, and compare it with the numerical simulation branch. The numerical simulation branch is obtained from the reduced amplitude SDE, whereas the theoretical branch is calculated from Equation (32). Additionally, to reflect the impact of stochastic perturbations on the system state, we plot the 50% and 95% quantile bands and obtain several sample branches through multiple simulations to demonstrate the range of random fluctuations (see Figure 10). When $\tau <\tau_{0}$, $Y_{mp}$ remains essentially near zero; when $\tau >\tau_{0}$, $Y_{mp}$ increases approximately linearly with τ, indicating that the system is more likely to exhibit oscillatory states with non-zero amplitude. At the same time, the theoretical branch matches the numerical simulation results well, indicating that the amplitude equation can accurately describe the variation trend of the most probable oscillation amplitude of the system under stochastic perturbations near the Hopf critical point. Furthermore, as τ exceeds $\tau_{0}$, the quantile band gradually expands, suggesting that after bifurcation, not only does the most probable oscillation amplitude increase, but the range of random fluctuations also widens, reflecting the increased uncertainty of the system.

A comprehensive analysis of Figure 8, Figure 9 and Figure 10 reveals that when the response delay of ants to changes in pheromone concentration is below a critical threshold, the foraging system tends to maintain stable coordination even under stochastic perturbations; however, once the response delay exceeds the critical value, the system is most likely to exhibit non-zero-amplitude oscillations. This indicates that the combined effects of pheromone feedback, ant behavioral transitions, and stochastic perturbations enhance the volatility of foraging flow, thereby making the oscillatory phenomenon induced by pheromone-mediated mixed feedback in the original model more pronounced under random conditions.

**Figure 11:**
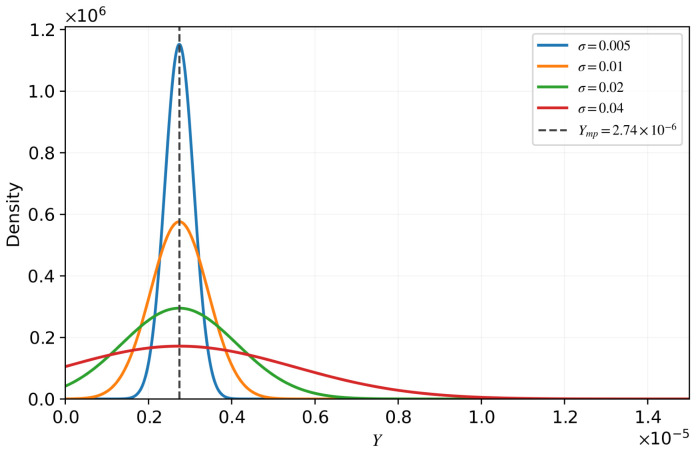
Stationary probability density of *Y* for different values of σ.

With a fixed response delay $\tau =7.551>\tau_{0}$, we select different noise intensities σ and plot the steady-state probability density distribution of the amplitude variable *Y* (see Figure 11). Within the present small-noise additive perturbation framework, as the noise intensity σ increases, the probability density curves gradually broaden and their peaks decrease, indicating that stronger stochastic perturbations increase the range of fluctuations in the system’s oscillation amplitude. At the same time, the peak positions of the probability density curves generally remain near the theoretical most probable value $Y_{mp}$, suggesting that, under a fixed response delay and within the present small-noise additive perturbation framework, noise intensity primarily affects the dispersion of the amplitude distribution. This implies that external stochastic perturbations, such as individual behavioral differences or environmental fluctuations, further amplify the random fluctuations in foraging flow, the proportion of foraging ants, and the pheromone feedback process.

### 4.4. Reconstruction in Original Variables

**Figure 12:**
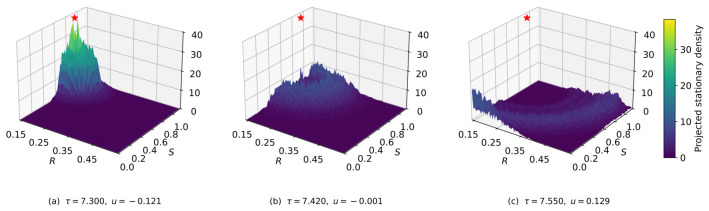
Reconstructed steady-state probability distributions in the $\left(R,S\right)$ plane obtained by projecting the reduced amplitude process along the dominant mode: (**a**) τ=7.300, u=−0.121. (**b**) τ=7.420, u=−0.001. (**c**) τ=7.550, u=0.129. The red asterisks indicate the system’s equilibrium position on the $\left(R,S\right)$ plane.

To establish a connection between the reduced-order amplitude model and the original system, we further project the reduced-order amplitude process back onto the original system’s $\left(R,S\right)$ plane along the direction of the dominant mode at the Hopf critical point. This yields the steady-state probability distribution structures of the receiver ant ratio *R* and the supplier ant ratio *S* under different response delays, as shown in Figure 12. It should be emphasized that Figure 12 is a visualization reconstructed from the reduced amplitude dynamics, aiming to connect the stochastic reduction theory with the original state variables, rather than a direct two-dimensional density estimate from simulations of the full stochastic delayed system. Here, the location and shape of the probability density peaks reflect the primary distribution characteristics of the system’s state fluctuations around the equilibrium under stochastic perturbations.

The results show that, before bifurcation, the probability density is primarily concentrated near the equilibrium point; near the critical point, the distribution widens significantly and exhibits an arc-shaped structure; whereas after bifurcation, the regions of high probability density are distributed along the closed orbits, manifesting as a noisy limit-cycle-like structure. This indicates that the stochastic P-bifurcation in the amplitude equation of this paper is not merely a mathematical reduction, but rather manifests as a shift in the probability distribution structure of ant foraging flow and food-transfer dynamics at the level of the original system variables.

**Figure 13:**
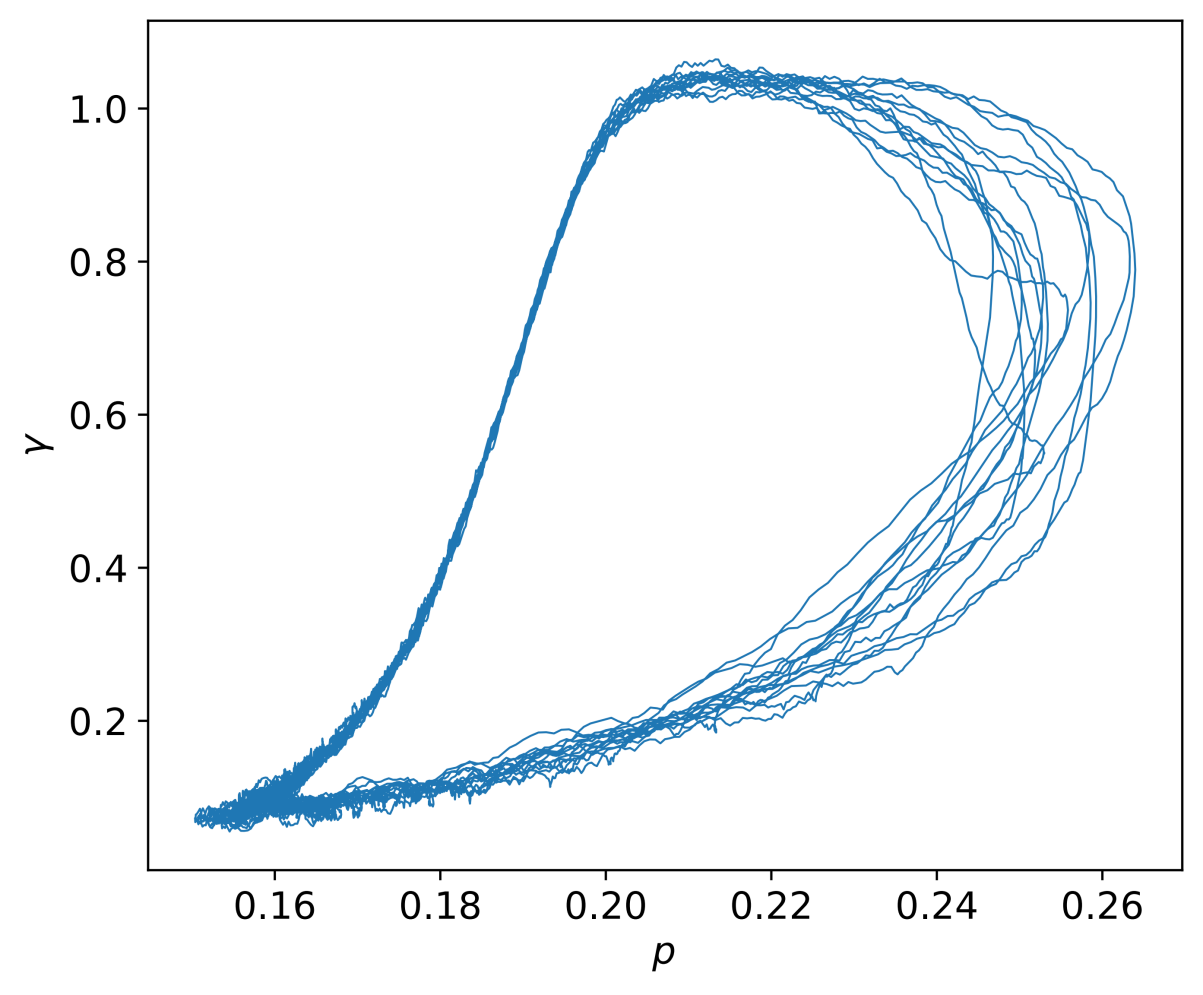
Phase trajectory of the system in the $\left(p,\gamma \right)$ plane at τ=7.55.

**Figure 14:**
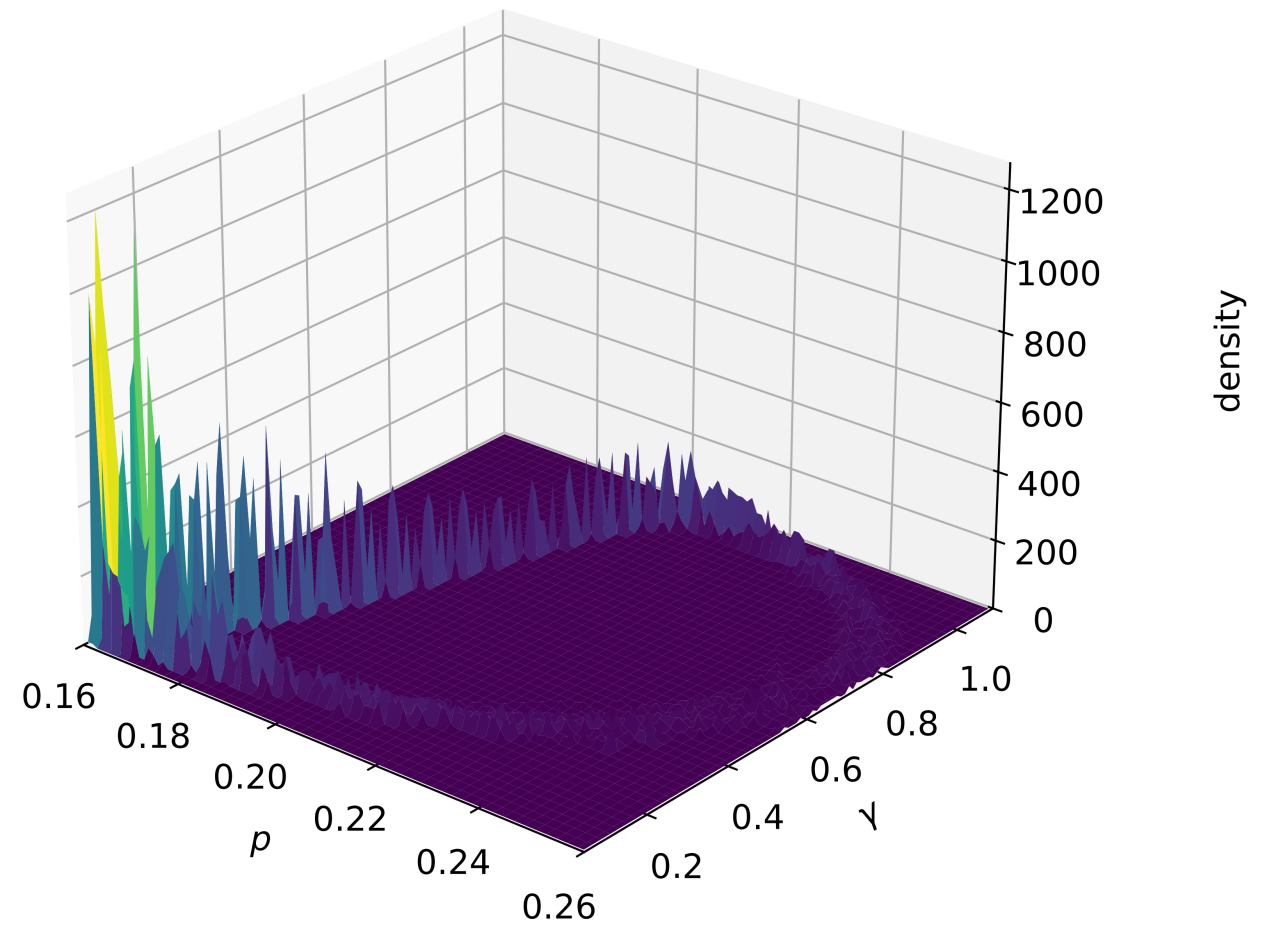
Joint steady-state probability density distribution of $\left(p,\gamma \right)$ at τ=7.55.

To further elucidate the manifestation of the pheromone feedback mechanism in the original system variables following a stochastic P-bifurcation, we set $\tau =7.55>\tau_{0}$ and plot the phase trajectories of the pheromone concentration *p* and the return rate γ, along with their joint steady-state probability density distribution, as shown in Figure 13 and Figure 14. As can be seen from the phase diagram, when τ=7.55, the system trajectories form a distinct closed orbit in the $\left(p,\gamma \right)$ plane, indicating that the pheromone concentration and return rate no longer tend toward a stationary equilibrium but instead exhibit continuous periodic fluctuations. Figure 14 shows that the probability density is distributed near the phase trajectories, indicating that once the response delay exceeds the critical value, a significant phase lag arises between pheromone feedback and ant return behavior, thereby sustaining a stochastic oscillatory structure. That is, the combined effects of positive feedback from pheromone deposition and negative feedback from pheromone evaporation cause the ant foraging system to exhibit sustained oscillations after the critical delay is exceeded.

Therefore, the stochastic P-bifurcation detected in the reduced amplitude equation is not merely a mathematical artifact. It corresponds to a structural transition of the probability distribution in the original ant-foraging variables, from fluctuations concentrated near the coexistence equilibrium to a noisy limit-cycle-like distribution induced by delayed pheromone feedback.

### 4.5. Entropy-Based Quantification of Uncertainty and Distributional Transitions

**Figure 15:**
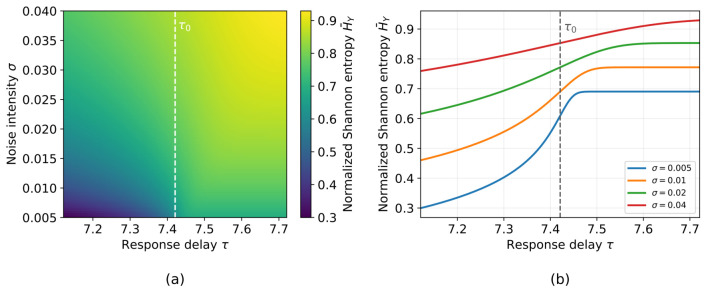
Normalized Shannon entropy of the stationary amplitude distribution $p_{st}\left(Y\right)$. (**a**) Entropy map in the (τ,σ) parameter plane. (**b**) Entropy variation with respect to the response delay τ for selected noise intensities.

Based on the steady-state amplitude distribution entropy defined in Section 3, we will now further examine, from a numerical perspective, how the response delay τ and noise intensity σ affect the uncertainty of the system’s probability distribution. Since the numerical comparisons are performed on a fixed discrete grid, we normalize the Shannon entropy to obtain

(33) $${\bar{H}}_{Y}\left(\tau ,\sigma \right)=-\frac{1}{\ln N_{bin}}\sum_{i=1}^{N_{bin}}P_{i}\left(\tau ,\sigma \right)\ln P_{i}\left(\tau ,\sigma \right).$$

where $P_{i}\left(\tau ,\sigma \right)$ denotes the probability mass in the *i*-th grid interval, $N_{\mathrm{bin}}$ is the total number of discrete bins, and $\ln N_{\mathrm{bin}}$ is used as the normalization factor. Figure 15 shows the normalized Shannon entropy of the steady-state distribution of the squared amplitude for different response delays τ and noise intensities σ. The dashed vertical line denotes the Hopf threshold $\tau_{0}$. As shown in Figure 15a, the entropy value increases with increasing response delay τ and noise intensity σ, indicating that in the distribution of reduced-order amplitudes, both τ and σ can alter the entropy and that greater delay and stronger random perturbations lead to a broader steady-state amplitude distribution. Figure 15b shows that the noise intensity σ determines the overall entropy level; as σ increases, the degree of diffusion in the probability distribution also increases. Furthermore, the region near the critical value $\tau_{0}$ is the most sensitive to changes in entropy. Once τ is sufficiently exceeded, ${\bar{H}}_{Y}$ tends to level off, a phenomenon that is particularly pronounced under weak noise conditions. Therefore, the reduced amplitude entropy provides a quantitative measure of the broadening of the stochastic oscillation amplitude distribution near the P-bifurcation threshold.

To examine whether this uncertainty expansion is reflected in the original variables, we also compute the normalized joint Shannon entropy of the empirical steady-state distributions in the (R,S) and (p,γ) planes. However, the absolute joint entropy of the original variables is strongly affected by the overall noise-induced dispersion and is less sensitive to the delay-induced geometric reorganization of the distribution. Therefore, instead of emphasizing the absolute entropy level, we consider entropy increments relative to the reference delay before the Hopf bifurcation. Under the condition of a fixed noise intensity σ, we define

(34) $$\begin{array}{cc} \Delta {\bar{H}}_{Y}\left(\tau ,\sigma \right) & ={\bar{H}}_{Y}\left(\tau ,\sigma \right)-{\bar{H}}_{Y}\left(\tau_{\mathrm{ref}},\sigma \right);\\ \Delta {\bar{H}}_{RS}\left(\tau ,\sigma \right) & ={\bar{H}}_{RS}\left(\tau ,\sigma \right)-{\bar{H}}_{RS}\left(\tau_{\mathrm{ref}},\sigma \right);\\ \Delta {\bar{H}}_{p\gamma }\left(\tau ,\sigma \right) & ={\bar{H}}_{p\gamma }\left(\tau ,\sigma \right)-{\bar{H}}_{p\gamma }\left(\tau_{\mathrm{ref}},\sigma \right). \end{array}$$

where, $\tau_{\mathrm{ref}}<\tau_{0}$ denotes the reference delay prior to the Hopf bifurcation. In numerical calculations, $\tau_{\mathrm{ref}}$ is set to the delay grid point closest to 7.2.

**Figure 16:**
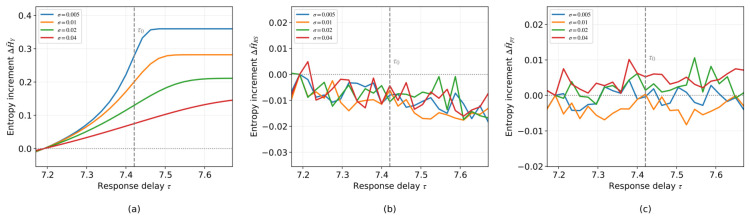
Increment in steady-state entropy relative to the reference delay before the Hopf bifurcation. (**a**) Reduced-order amplitude distribution $Y=\rho^{2}$; (**b**) Joint distribution of the original system variables (R,S); (**c**) Joint distribution of the original system variables (p,γ).

Figure 16 illustrates the entropy increments relative to the reference delay before the Hopf bifurcation. The reduced-order amplitude entropy increment $\Delta {\bar{H}}_{Y}$ exhibits a clear increase near and beyond $\tau_{0}$, indicating that the stochastic amplitude distribution becomes more dispersed after the onset of delay-induced oscillations. In contrast, the entropy increments of the original-variable distributions, $\Delta {\bar{H}}_{RS}$ and $\Delta {\bar{H}}_{p\gamma }$, remain much smaller and fluctuate around zero. This difference suggests that Shannon entropy primarily quantifies the degree of probabilistic diffusion rather than the geometric reorganization of the distribution. Hence, entropy is effective for measuring uncertainty expansion in the reduced amplitude variable, but it is not sufficient to characterize structural transitions in the projected distributions of the original state variables.

Since Shannon entropy does not directly quantify changes in distributional geometry, we further employ the normalized Jensen–Shannon divergence to measure the distributional distance from the pre-Hopf reference distribution. Here, the Jensen–Shannon divergence is not used as an additional criterion for the occurrence of P-bifurcation. Rather, once the delay-induced transition has occurred, it provides a quantitative measure of how far the stationary distribution has moved away from its below-threshold probabilistic organization. For each fixed noise intensity σ, the empirical steady-state distribution at a representative pre-Hopf delay $\tau_{\mathrm{ref}}<\tau_{0}$ is used as the reference distribution, rather than a deterministic limit-cycle distribution. This reference delay is selected because it lies in the stable below-threshold regime and represents the baseline probabilistic organization before the delay-induced transition. Let $P\left(\tau ,\sigma \right)$ denote the empirical steady-state distribution under the parameter pair $\left(\tau ,\sigma \right)$, and let $P_{ref}\left(\sigma \right)=P\left(\tau_{ref},\sigma \right)$. The Jensen–Shannon divergence is defined as

(35) $$D_{JS}\left(P∥Q\right)=\frac{1}{2}D_{KL}\left(P∥M\right)+\frac{1}{2}D_{KL}\left(Q∥M\right),M=\frac{1}{2}\left(P+Q\right).$$

All empirical distributions are computed on a common support using the same binning scheme. Therefore, the Jensen–Shannon divergence remains finite even when the distribution changes from a unimodal to a multimodal form, although the detailed modal structure should be interpreted together with the density plots rather than from the divergence value alone.

When the natural logarithm is used, the normalized Jensen–Shannon divergence is defined as

(36) $${\bar{D}}_{JS}\left(P\|Q\right)=\frac{D_{JS}\left(P\|Q\right)}{\ln2},{\bar{D}}_{JS}\in \left[0,1\right].$$

Then we define three types of indicators:

(37) $$\begin{array}{cc} {\bar{JS}}_{Y}\left(\tau ,\sigma \right) & ={\bar{D}}_{JS}\left(P_{Y}\left(\tau ,\sigma \right)∥P_{Y}\left(\tau_{\mathrm{ref}},\sigma \right)\right);\\ {\bar{JS}}_{RS}\left(\tau ,\sigma \right) & ={\bar{D}}_{JS}\left(P_{RS}\left(\tau ,\sigma \right)∥P_{RS}\left(\tau_{\mathrm{ref}},\sigma \right)\right);\\ {\bar{JS}}_{p\gamma }\left(\tau ,\sigma \right) & ={\bar{D}}_{JS}\left(P_{p\gamma }\left(\tau ,\sigma \right)∥P_{p\gamma }\left(\tau_{\mathrm{ref}},\sigma \right)\right). \end{array}$$

**Figure 17:**
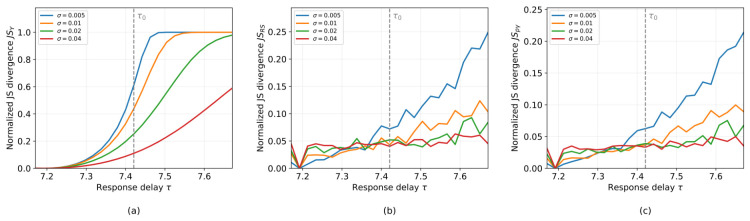
Normalized Jensen–Shannon divergence relative to the steady-state distribution of the reference delay prior to the Hopf bifurcation. (**a**) Reduced-order amplitude distribution $Y=\rho^{2}$; (**b**) Joint distribution of the original system variables (R,S); (**c**) Joint distribution of the original system variables (p,γ).

Figure 17 was obtained by calculating the Jensen–Shannon divergence between the steady-state distribution at each response delay τ and the corresponding reference distribution prior to the Hopf critical point at $\tau_{\mathrm{ref}}$, and then normalizing the result. It can be seen that the JS divergence of the reduced-order amplitude distribution increases rapidly as τ approaches and exceeds $\tau_{0}$, indicating a pronounced departure from the below-threshold reference amplitude distribution. Compared with the entropy increment, this result more directly quantifies the structural displacement of probability mass from the neighborhood of zero amplitude toward nonzero stochastic oscillation amplitudes. At the same time, the JS divergences of the original-variable distributions also increase after the Hopf threshold, although their magnitudes are smaller and their variations are more gradual. This is because the two-dimensional projections in the original variables are affected simultaneously by delay-induced reorganization and noise-induced smoothing. These results indicate that the response delay primarily drives the structural departure from the below-threshold probabilistic organization, whereas the noise intensity mainly controls distributional diffusion and may smooth the contrast between below-threshold and above-threshold distributions. In other words, response delay does not merely amplify random fluctuations; it changes the probabilistic organization of the pheromone-mediated feedback loop among foragers, suppliers, receivers, and returning ants. Therefore, the JS divergence complements the entropy analysis by converting the qualitative notion of distributional reorganization into a bounded quantitative distance.

To further summarize the entropy-based uncertainty measures and the distributional-distance indicators, representative values relative to the reference delay before the Hopf bifurcation are listed in Table 3. Here, the entropy increments are computed between the above-threshold delay and the reference delay $\tau_{\mathrm{ref}}<\tau_{0}$, while the normalized Jensen–Shannon divergences quantify the structural distance between the corresponding probability distributions. This table provides a compact quantitative comparison of the reduced amplitude distribution, the behavioral-state distribution in the (R,S) plane, and the pheromone feedback distribution in the (p,γ) plane. The numerical settings used for the entropy and distributional-distance indicators in Figure 15, Figure 16 and Figure 17 and Table 3 are summarized in Table A2.

Table 3 further summarizes the entropy increments and normalized JS divergences between the above-threshold state and the reference state before the Hopf bifurcation. The positive values of $\Delta {\bar{H}}_{Y}$ indicate that the reduced amplitude distribution becomes more uncertain after the transition, whereas the small values of $\Delta {\bar{H}}_{RS}$ and $\Delta {\bar{H}}_{p\gamma }$ show that entropy changes in the original-variable projections are relatively weak. In contrast, the nonzero JS divergences provide a direct quantitative measure of the distributional distance from the below-threshold reference state. Thus, the role of JS divergence is not to prove the occurrence of the bifurcation but to quantify the magnitude of probabilistic reorganization induced by the response delay.

Overall, the entropy and divergence results provide a biological interpretation of the stochastic oscillations in the delayed pheromone-feedback system. A low-entropy distribution corresponds to a predictable foraging regime near the stable feedback equilibrium, whereas a higher entropy indicates broader variability in collective foraging amplitudes caused by noise. In contrast, a larger Jensen–Shannon divergence reflects a structural shift of the probability distribution induced by response delay. Thus, noise mainly broadens the range of possible foraging activity levels, while delay changes the dominant regulatory mode of the colony.

## 5. Conclusions

This paper establishes a feedback-based foraging model for ants that incorporates response time delays and Gaussian white noise and investigates the stochastic dynamical behavior of the system and the mechanisms underlying the evolution of its probabilistic structure under the combined effects of time delays and random perturbations. The results indicate that response delay is the primary mechanism driving the system’s transition from a stable state to an oscillatory state. When the delay exceeds a critical threshold, the system loses its original stability and enters sustained oscillation via a Hopf bifurcation. This suggests that the time delay between pheromone feedback and the ants’ response to return to the nest disrupts the original stable foraging rhythm and induces periodic fluctuations.

Subsequently, by extending the study to a stochastic environment, we find that noise intensity primarily influences the degree of diffusion and the level of uncertainty in the probability distribution under oscillatory conditions. As noise increases, the steady-state probability density gradually broadens, and its peak gradually decreases, indicating that stochastic perturbations primarily promote the expansion of uncertainty in the oscillation amplitude, while their impact on the location of the most probable oscillation amplitude is relatively limited.

Building on this, we introduce entropy to further quantify the uncertainty in the stochastic transition process. In particular, normalized Shannon entropy characterizes the expansion of uncertainty in the distribution of oscillation amplitudes near the Hopf critical region, while the Jensen–Shannon divergence clearly reveals the phenomenon of probabilistic structural reorganization induced by response delays. In biological terms, entropy growth reflects reduced predictability of collective foraging, while Jensen–Shannon divergence captures the delay-induced shift from stable feedback regulation to oscillatory foraging. These results suggest that traditional bifurcation analysis alone is insufficient to describe the distributional evolution of time-delay stochastic foraging systems and that information-theoretic metrics provide a distributional-level characterization of uncertainty expansion and probabilistic reorganization.

Based on the above results, we conclude that when ants can respond promptly to changes in pheromone concentration, pheromone feedback helps maintain coordination among foraging ants, supplier ants, and receiver ants; however, when the response delay exceeds a certain critical threshold, the phase difference between pheromone feedback and behavioral transitions induces sustained oscillations, which, under the influence of random perturbations, further amplify the system’s instability.

These findings explain the stability regulation mechanisms of ant foraging systems in stochastic environments from a combined dynamical and information-theoretic perspective and provide a theoretical framework for studying time-delay feedback, stochastic perturbations, and the evolution of probabilistic structures in swarm intelligence systems. Future research may further explore non-Gaussian noise, state-dependent noise, multi-delay feedback, and stochastic swarm dynamics under complex network structures.

## Figures and Tables

**Table 1:** Parameter list.

Parameter	Value	Parameter	Value
M	1	N	1
ν	0.45	μ	0.2
α	0.05	β	0.3
$\gamma_{0}$	0.05	$\tau_{\gamma }$	1
κ	1	$p_{0}$	0.2

**Table 2:** Numerical verification of H(1), H(2), and the Hopf threshold.

Quantity	Numerical Value	Purpose
L(0.2)	−0.028505<0	Verification of H(1)
Positive roots of L(x)=0	$x_{1}=0.004288,\;x_{2}=0.425064$	Critical frequencies
Corresponding frequencies	$\omega_{1}=0.065480,\;\omega_{2}=0.651969$	$\omega_{i}=\sqrt{x_{i}}$
$\left(S_{i},C_{i}\right)$ for $\omega_{2}=0.651969$	(−0.992087,0.125553)	Computation of $\theta_{i}$
$\theta_{i}=\mathrm{atan}2\left(S_{i},C_{i}\right)$	4.838274	Phase angle
First critical delay	$\tau_{0}=7.421015$	Hopf threshold
Other positive candidate delay	11.604825	Larger than $\tau_{0}$
*I* in H(2)	$2.0108\times 10^{-4}\neq 0$	Verification of H(2)
Redλ/dττ=τ0	0.012123≠0	Transversality condition

**Table 3:** Entropy increments and normalized distributional-distance indicators relative to the reference delay before the Hopf bifurcation.

σ	$\Delta {\bar{H}}_{Y}$	$\Delta {\bar{H}}_{\mathrm{RS}}$	$\Delta {\bar{H}}_{p\gamma }$	${\bar{\mathrm{JS}}}_{Y}$	${\bar{\mathrm{JS}}}_{\mathrm{RS}}$	${\bar{\mathrm{JS}}}_{p\gamma }$
0.005	0.359651	−0.013354	−0.001992	1.000000	0.129122	0.115050
0.010	0.281630	−0.014774	−0.003918	0.992897	0.082065	0.067151
0.020	0.200232	−0.000879	0.010595	0.723902	0.055772	0.041895
0.040	0.117207	−0.009196	0.003122	0.308684	0.047581	0.038835

## Data Availability

The original contributions presented in this study are included in the article. Further inquiries can be directed to the corresponding author.

## Abbreviations

The following abbreviations are used in this manuscript:

DDE	Delay differential equation
SDE	Stochastic differential equation
ODE	Ordinary differential equation
PDE	Partial differential equation
PDE–ODE	Partial differential equation–ordinary differential equation
JS	Jensen–Shannon
KL	Kullback–Leibler
PDF	Probability density function
MC	Monte Carlo

## Appendix A

### Appendix A.1

Explicit expressions of the adjoint eigenvector coefficients.

$$\begin{array}{cc} \alpha_{1}= & \frac{\left[-\beta \frac{N}{M}S^{*}\nu \chi_{3}-\nu \beta NS^{*}\chi_{2}\right]\chi_{4}}{D_{\mathrm{adj}}^{\;2}}-\frac{\omega_{0}^{2}\left(\chi_{1}+\chi_{2}\right)\nu \beta NS^{*}}{D_{\mathrm{adj}}},\\ \beta_{1}= & -\frac{\omega_{0}\nu \beta NS^{*}\chi_{4}}{D_{\mathrm{adj}}}-\frac{\left[-\beta \frac{N}{M}S^{*}\nu \chi_{3}-\nu \beta NS^{*}\chi_{2}\right]\omega_{0}\left(\chi_{1}+\chi_{2}\right)}{D_{\mathrm{adj}}},\\ \alpha_{2}= & \frac{\left[-\beta \frac{M}{N}R^{*}\nu \beta NS^{*}-\chi_{1}\nu \chi_{3}\right]\chi_{4}}{D_{\mathrm{adj}}^{\;2}}-\frac{\omega_{0}^{2}\nu \chi_{3}\left(\chi_{1}+\chi_{2}\right)}{D_{\mathrm{adj}}},\\ \beta_{2}= & -\frac{\omega_{0}\nu \chi_{3}\chi_{4}}{D_{\mathrm{adj}}}-\frac{\left[-\beta \frac{M}{N}R^{*}\nu \beta NS^{*}-\chi_{1}\nu \chi_{3}\right]\omega_{0}\left(\chi_{1}+\chi_{2}\right)}{D_{\mathrm{adj}}},\\ \alpha_{4}= & -\frac{-\mu \cos\left(\omega_{0}\tau \right)-\omega_{0}\sin\left(\omega_{0}\tau \right)}{f^{\prime }\left(p^{*}\right)/\tau_{\gamma }},\\ \beta_{4}= & \frac{-\mu \sin\left(\omega_{0}\tau \right)-\omega_{0}\cos\left(\omega_{0}\tau \right)}{f^{\prime }\left(p^{*}\right)/\tau_{\gamma }}. \end{array}$$

where

$$\begin{array}{cc} \chi_{1}= & -\alpha -\beta \frac{M}{N}S^{*},\\ \chi_{2}= & -\gamma^{*}-\beta \frac{N}{M}R^{*},\\ \chi_{3}= & -\gamma^{*}M+\beta NR^{*},\\ \chi_{4}= & \chi_{1}\chi_{2}-\beta^{2}R^{*}S^{*}-\omega_{0}^{2},\\ D_{\mathrm{adj}}= & \chi_{4}^{2}+{\left[\omega_{0}\left(\chi_{1}+\chi_{2}\right)\right]}^{2}. \end{array}$$

### Appendix A.2

The detailed numerical procedure used to obtain the critical delay $\tau_{0}=7.421015$ reported in Section 4 is given below.

For the parameter values in Table 1, solving Equation (2) gives

$$E^{*}=\left(R^{*},\;S^{*},\;p^{*},\;\gamma^{*}\right)=\left(0.159511,\;0.878191,\;0.189110,\;0.345004\right).$$

Since

$$f\left(p\right)=\frac{\kappa }{1+\exp[-\eta \left(p-p_{0}\right)]},$$

we have

$$f^{\prime }\left(p\right)=\frac{\kappa \eta \exp[-\eta \left(p-p_{0}\right)]}{{\left(1+\exp[-\eta \left(p-p_{0}\right)]\right)}^{2}}.$$

$$\begin{array}{cc} f^{\prime }\left(p^{*}\right) & =16.638131,\;b_{43}=\frac{f^{\prime }\left(p^{*}\right)}{\tau_{\gamma }}=16.638131. \end{array}$$

According to the definitions of $m_{i}$, $n_{i}$, and $d_{i}$ in Section 2, we obtain

$$\begin{array}{cc} \left(m_{1},\;m_{2},\;m_{3},\;m_{4}\right) & =(-1.906315,\;1.158115,\;0.273907,\;0.022107),\\ \left(n_{1},\;n_{2},\;n_{3}\right) & =(0.054814,\;-0.022428,\;-0.000262),\\ \left(d_{1},\;d_{2},\;d_{3},\;d_{4}\right) & =(1.317807,\;-0.490606,\;-0.107467,\;0.000470). \end{array}$$

Hence,

$$L\left(x\right)=x^{4}+1.317807x^{3}-0.490606x^{2}-0.107467x+0.000470.$$

Since

L(0.2)=−0.028505<0,

assumption H(1) is satisfied.

Solving L(x)=0 gives two positive roots:

$$x_{1}=0.004288,\;x_{2}=0.425064.$$

Therefore,

$$\omega_{1}=\sqrt{x_{1}}=0.065480,\;\omega_{2}=\sqrt{x_{2}}=0.651969.$$

For $\omega_{1}=0.065480$, Equations (8) and (9) give

$$S_{1}=0.688834,\;C_{1}=0.724919,$$

and therefore

$$\theta_{1}=\mathrm{atan}2\left(S_{1},C_{1}\right)=0.759880,\;\tau_{1}^{\left(0\right)}=\frac{\theta_{1}}{\omega_{1}}=11.604825.$$

For $\omega_{2}=0.651969$, Equations (8) and (9) give

$$S_{2}=-0.992087,\;C_{2}=0.125553.$$

Taking $\theta_{2}\in \left[0,2\pi \right)$, we obtain

$$\theta_{2}=\mathrm{atan}2\left(S_{2},C_{2}\right)=4.838274,$$

and hence

$$\tau_{2}^{\left(0\right)}=\frac{\theta_{2}}{\omega_{2}}=\frac{4.838274}{0.651969}=7.421015.$$

Since

$$\tau_{2}^{\left(0\right)}=7.421015<\tau_{1}^{\left(0\right)}=11.604825,$$

the first positive critical delay is

$$\tau_{0}=7.421015,\;\omega_{0}=0.651969.$$

Finally, substituting $\omega_{0}=0.651969$ into H(2) gives

I=0.000201≠0.

Equivalently,

Redλdτλ=iω0,τ=τ0=0.012123≠0.

Thus, the transversality condition holds, and the Hopf bifurcation occurs at

$$\tau =\tau_{0}=7.421015.$$

## Appendix B

**Table A1:** Detailed numerical settings for the simulations in Section 4.

Item	Value or Description
Logistic feedback parameters	$\kappa =1,\;p_{0}=0.2,\;\eta =80$.
Noise intensity	σ=0 for Figure 4 and Figure 5; σ=0.02 for Figure 6 and Figure 7; σ=0.01 for Figure 8, Figure 9 and Figure 10 and Figure 12, Figure 13 and Figure 14; σ=0.005,0.01,0.02,0.04 for Figure 11.
Time step	Δt=0.05 for Figure 4, Figure 5, Figure 6 and Figure 7 and Δt=0.01 for Figure 13 and Figure 14. For Figure 8, Figure 9 and Figure 10 and Figure 12, a small numerical step of order $10^{-3}$ is used.
Total simulation time	T=3000 for Figure 4 and Figure 5; *T* = 16,000 for Figure 6 and Figure 7; T=2500 for Figure 13 and Figure 14.
Initial history function	$X\left(\theta \right)=E^{*}+\delta ,\;\theta \in \left[-\tau ,0\right]$, where δ is a small constant perturbation.
Delay discretization	The delayed pheromone term p(t−τ) is evaluated on the numerical grid. Linear interpolation is used when τ/Δt is not an integer.
Transient removal	The first 25% of each reduced-amplitude trajectory is discarded in Figure 8, Figure 9 and Figure 12, and the first 35% of the trajectory is discarded in Figure 10. For Figure 13 and Figure 14, the first $T_{\mathrm{trans}}=1000$ out of T=2500 is discarded.
Number of sample paths	Figure 4, Figure 5, Figure 6 and Figure 7, Figure 13 and Figure 14 use representative trajectories. For Figure 10, 10 independent sample paths are used for each value of τ, and 5 representative sample branches are displayed.
Density estimation	Histogram-based density estimation is used for Figure 8, Figure 9 and Figure 10, Figure 12 and Figure 14. Figure 11 is obtained from the theoretical stationary density of the reduced amplitude variable.

**Table A2:** Numerical settings for entropy and distributional-distance indicators.

Item	Setting
Reduced amplitude entropy	The entropy of the reduced amplitude variable $Y=\rho^{2}$ is computed from a fixed one-dimensional discrete probability distribution. The normalized Shannon entropy is calculated using $\ln K_{Y}$ as the normalization factor.
Original-variable entropy	The empirical joint entropies of (R,S) and (p,γ) are computed from two-dimensional histograms with 70×70 bins. The same histogram ranges are used for all values of τ and σ.
Entropy normalization	All entropy indicators are normalized by the logarithm of the number of discrete grid cells, so that the resulting values are comparable across different distributions. Zero-probability terms are treated according to 0ln0=0.
Jensen–Shannon divergence	The normalized Jensen–Shannon divergence is computed as ${\bar{D}}_{JS}=D_{JS}/\ln2$. The compared probability distributions are evaluated on the same discrete grid.
Reference delay	The reference delay before the Hopf bifurcation is chosen as the grid point closest to 7.20, namely $\tau_{\mathrm{ref}}=7.191848<\tau_{0}$. The same reference delay is used for entropy increments and Jensen–Shannon divergence calculations.
Above-threshold delay for Table 3	The above-threshold delay is chosen as the grid point closest to 7.55, namely $\tau_{+}=7.546015>\tau_{0}$.
Monte Carlo simulations	For the original-variable distributions, $N_{\mathrm{rep}}=5$ Monte Carlo realizations are used for each parameter pair. The total simulation time is T=2500, the transient time is $T_{\mathrm{trans}}=1000$, the time step is Δt=0.01, and the downsampling interval is 10.
Parameter grid	The delay is sampled on $\left[\tau_{0}-0.25,\tau_{0}+0.25\right]$ using 25 uniformly spaced grid points. The noise intensities are σ=0.005,0.010,0.020, and 0.040.

## References

[1] Gordon D.M. (2019). The Ecology of Collective Behavior in Ants. Annu. Rev. Entomol..

[2] Fernández-López P., Oro D., Lloret-Cabot R., Genovart M., Garriga J., Bartumeus F. (2025). Foraging ants as liquid brains: Movement heterogeneity shapes collective efficiency. Proc. Natl. Acad. Sci. USA.

[3] Lecheval V., Larson H., Burns D.D.R., Ellis S., Powell S., Donaldson-Matasci M.C., Robinson E.J.H. (2021). From foraging trails to transport networks: How the quality-distance trade-off shapes network structure. Proc. R. Soc. B Biol. Sci..

[4] Hartman S., Ryan S.D., Karamched B.R. (2024). Walk this way: Modeling foraging ant dynamics in multiple food source environments. J. Math. Biol..

[5] Sepulchre R., Drion G., Franci A. (2019). Control Across Scales by Positive and Negative Feedback. Annu. Rev. Control Robot. Auton. Syst..

[6] Das A., Chaffey T., Sepulchre R. (2022). Oscillations in mixed-feedback systems. Syst. Control Lett..

[7] Czaczkes T.J., Heinze J. (2015). Ants adjust their pheromone deposition to a changing environment and their probability of making errors. Proc. R. Soc. B Biol. Sci..

[8] Porfiri M., Abaid N., Garnier S. (2024). Socially driven negative feedback regulates activity and energy use in ant colonies. PLoS Comput. Biol..

[9] Reina A., Marshall J.A.R. (2022). Negative feedback may suppress variation to improve collective foraging performance. PLoS Comput. Biol..

[10] Dussutour A., Beekman M., Nicolis S.C., Meyer B. (2009). Noise improves collective decision-making by ants in dynamic environments. Proc. R. Soc. B Biol. Sci..

[11] Rausch I., Reina A., Simoens P., Khaluf Y. (2019). Coherent collective behaviour emerging from decentralised balancing of social feedback and noise. Swarm Intell..

[12] Jeanson R., Dussutour A., Fourcassié V. (2012). Key Factors for the Emergence of Collective Decision in Invertebrates. Front. Neurosci..

[13] Abdenebaoui L., Kreowski H.J., Kuske S. (2021). A Graph-Transformational Approach to Swarm Computation. Entropy.

[14] Udiani O., Pinter-Wollman N., Kang Y. (2015). Identifying robustness in the regulation of collective foraging of ant colonies using an interaction-based model with backward bifurcation. J. Theor. Biol..

[15] Ryan S.D. (2016). A model for collective dynamics in ant raids. J. Math. Biol..

[16] Pagliara R., Gordon D.M., Leonard N.E. (2018). Regulation of harvester ant foraging as a closed-loop excitable system. PLoS Comput. Biol..

[17] Feng T., Qiu Z. (2021). Foraging dynamics of social insect colonies with resource constraints in random environments. Appl. Math. Lett..

[18] Feng T., Liu C. (2023). A mathematical framework for collective foraging behavior of social insect colonies in multi-dynamic environments. Appl. Math. Lett..

[19] Liu C., Feng T. (2024). Unraveling the forces shaping foraging dynamics in harvester ant colonies: Recruitment efficiency and environmental variability. Math. Biosci..

[20] Glass D.S., Jin X., Riedel-Kruse I.H. (2021). Nonlinear delay differential equations and their application to modeling biological network motifs. Nat. Commun..

[21] Yang R., Ma Y., Zhang C. (2021). Time delay induced Hopf bifurcation in a diffusive predator–prey model with prey toxicity. Adv. Differ. Equ..

[22] Zhang R., Liu X., Wei C. (2021). Stability and Hopf Bifurcation of a Delayed Mutualistic System. Int. J. Bifurc. Chaos.

[23] Cook C.N., Lemanski N.J., Mosqueiro T., Ozturk C., Gadau J., Pinter-Wollman N., Smith B.H. (2020). Individual learning phenotypes drive collective behavior. Proc. Natl. Acad. Sci. USA.

[24] Richardson T.O., Kay T., Braunschweig R., Journeau O.A., Rüegg M., McGregor S., De Los Rios P., Keller L. (2021). Ant behavioral maturation is mediated by a stochastic transition between two fundamental states. Curr. Biol..

[25] Bidari S., El Hady A., Davidson J.D., Kilpatrick Z.P. (2022). Stochastic dynamics of social patch foraging decisions. Phys. Rev. Res..

[26] Lecheval V., Robinson E.J.H., Mann R.P. (2024). Random walks with spatial and temporal resets can explain individual and colony-level searching patterns in ants. J. R. Soc. Interface.

[27] Dodoková K., Malíčková M., Yates C., Dussutour A., Bodova K. (2024). A stochastic model of ant trail formation and maintenance in static and dynamic environments. Swarm Intell..

[28] Gilpin C., Darmon D., Siwy Z., Martens C. (2018). Information Dynamics of a Nonlinear Stochastic Nanopore System. Entropy.

[29] Liang X.S. (2014). Entropy Evolution and Uncertainty Estimation with Dynamical Systems. Entropy.

[30] Lin J. (1991). Divergence measures based on the Shannon entropy. IEEE Trans. Inf. Theory.

[31] Valentine A., Gordon D.M., Bizyaeva A.S. Mixed-feedback oscillations in the foraging dynamics of arboreal turtle ants. Proceedings of the 64th IEEE Conference on Decision and Control.

[32] Jia W., Xu Y., Li D., Hu R. (2021). Stochastic Analysis of Predator–Prey Models under Combined Gaussian and Poisson White Noise via Stochastic Averaging Method. Entropy.

[33] Powanwe A.S., Longtin A. (2021). Amplitude-phase description of stochastic neural oscillators across the Hopf bifurcation. Phys. Rev. Res..

[34] Wang D., Li X., Zhu X., Zhang E. (2025). Complex dynamics for a discretized fractional-order predator-prey system with constant-yield prey harvesting and fear effect. Math. Model. Control.

[35] Cao Y., Guo P., Guerrini L. (2025). Dynamical behaviour of a two prey and one predator system with indirect effect and time delay. Alex. Eng. J..

[36] Tanweer S., Khasawneh F.A., Munch E., Tempelman J.R. (2024). A topological framework for identifying phenomenological bifurcations in stochastic dynamical systems. Nonlinear Dyn..

